# Genetic Diversity, Population Structure and Ancestral Origin of Australian Wheat

**DOI:** 10.3389/fpls.2017.02115

**Published:** 2017-12-12

**Authors:** Reem Joukhadar, Hans D. Daetwyler, Urmil K. Bansal, Anthony R. Gendall, Matthew J. Hayden

**Affiliations:** ^1^Department of Animal, Plant and Soil Sciences, La Trobe University, Bundoora, VIC, Australia; ^2^Agriculture Victoria Research, AgriBio, Centre for Agribioscience, Bundoora, VIC, Australia; ^3^School of Applied Systems Biology, La Trobe University, Bundoora, VIC, Australia; ^4^School of Life and Environmental Sciences, The University of Sydney Plant Breeding Institute, Cobbitty, NSW, Australia

**Keywords:** australian wheat, geographical ancestor, genetic diversity, *in-silico* chromosome painting, population structure, single nucleotide polymorphism

## Abstract

Since the introduction of wheat into Australia by the First Fleet settlers, germplasm from different geographical origins has been used to adapt wheat to the Australian climate through selection and breeding. In this paper, we used 482 cultivars, representing the breeding history of bread wheat in Australia since 1840, to characterize their diversity and population structure and to define the geographical ancestral background of Australian wheat germplasm. This was achieved by comparing them to a global wheat collection using *in-silico* chromosome painting based on SNP genotyping. The global collection involved 2,335 wheat accessions which was divided into 23 different geographical subpopulations. However, the whole set was reduced to 1,544 accessions to increase the differentiation and decrease the admixture among different global subpopulations to increase the power of the painting analysis. Our analysis revealed that the structure of Australian wheat germplasm and its geographic ancestors have changed significantly through time, especially after the Green Revolution. Before 1920, breeders used cultivars from around the world, but mainly Europe and Africa, to select potential cultivars that could tolerate Australian growing conditions. Between 1921 and 1970, a dependence on African wheat germplasm became more prevalent. Since 1970, a heavy reliance on International Maize and Wheat Improvement Center (CIMMYT) germplasm has persisted. Combining the results from linkage disequilibrium, population structure and *in-silico* painting revealed that the dependence on CIMMYT materials has varied among different Australian States, has shrunken the germplasm effective population size and produced larger linkage disequilibrium blocks. This study documents the evolutionary history of wheat breeding in Australia and provides an understanding for how the wheat genome has been adapted to local growing conditions. This information provides a guide for industry to assist with maintaining genetic diversity for long-term selection gains and to plan future breeding programs.

## Introduction

The history of Australian wheat began when the First Fleet arrived in Sydney in 1788. Facing a different environment to their home country, their major challenge was to yield sufficient grain to cover their needs (PWC, 2011). Initially, farmers introduced cultivars from the UK and different European countries to test under Australian conditions; later efforts involved wheat from other regions. The first documented attempt to improve wheat for Australian growing conditions was in 1860 when a farmer selected rust free plants, subsequently released as Purple Straw, in a heavily rust-infected field (Henzell, 2007) from the Italian wheat Tuscan (Quirk, 1982; Marino, 2012). Subsequent selections from Purple Straw and other dominant cultivars grown at the time, like Ward's Prolific, led to several cultivars that remained important into the first decade of the twentieth century (Spennemann, 2001).

Although the biology of plant reproduction and its potential for crop improvement was well understood at the time of the First Fleet, the initial attempt to improve wheat through cross-breeding did not occur until 1889 with the work of William James Farrer. His pioneering work started by identifying morphological, agronomic and physiological characters required to grow wheat under Australian conditions while maintaining good milling and baking quality (Lupton, 1987). This enabled Farrer to make better decisions for selecting appropriate parents to hybridize. The cultivar Federation released in 1901 was one of his most successful attempts. He developed Federation by crossing the early maturing Indian landrace Etawah, which escaped rust and drought, with the high milling and baking quality Canadian cultivar Fife and the adapted cultivar Purple Straw. One of the main outcomes of Farrer's pioneering work was the expansion of wheat cultivation into parts of the country considered unsuitable for wheat production (Wenholz, 1930). In 1945, the efforts of Walter Lawry Waterhouse lead to the release of cultivar the Gabo, which dominated the country and was later adopted by countries such as Mexico for its high yield (Henzell, 2007). Since the 1960s, semi-dwarf materials from the International Maize and Wheat Improvement Center (CIMMYT) have been widely used in Australian wheat breeding; their derived varieties occupied approximately 98% of the wheat growing area in 2003 (Brennan and Quade, 2006) and their dominance continues today.

The evolution of Australian wheat germplasm is defined by two major events. First, Farrer's introduction of earlier flowering materials at the beginning of the twentieth century, and second, when CIMMYT semi-dwarf germplasm dominated the planting area after 1970 (Pugsley, 1983). These events underlined major changes to the structure of the Australian germplasm. Previous reports based on SSR and RFLP markers also indicate that the use of CIMMYT semi-dwarf wheat had a major impact on the structure of Australian wheat germplasm (Paull et al., 1998; Parker et al., 2002).

Although the major events in the evolution of Australian wheat are well documented by pedigree information, such records are limited by cultivar selection bias that occurred during variety development, inaccurate or incomplete pedigrees, and background relatedness (Bernardo, 1996; Barrett et al., 1998). Even when pedigree records are identical among individuals, large variability of the identical-by-decent genomic proportion was observed (Hill and Weir, 2011; Allendorf, 2017). While accurate pedigree data provides a quantitative prediction of whole germplasm constitution, this prediction is an overall mean for the whole genome and cannot be traced to a particular genomic locus or region of DNA. Moreover, pedigree records do not take into consideration any identical-by-descent relations prior to the first recorded hybridization. In contrast, molecular genotyping technologies (e.g., single nucleotide polymorphisms (SNPs) allied with advanced population genetic statistical tools provide an alternative and more accurate approach to understand the ancestral origin of germplasm (Lawson et al., 2012).

There are two means to infer ancestral populations: global and local. Global ancestry estimates the contribution of each ancestral population as a proportion of each individual's whole genome, while local ancestry assigns individual chromosomes as a mosaic of fragments originating from different ancestral populations (Padhukasahasram, 2014). The Inference of ancestral origin has been extensively used for many applications including to understand immigration (Arauna et al., 2017), expansion (Lesser et al., 2013), natural selection (Jin et al., 2012), and admixture history of populations (Busby et al., 2015). It has also been used for mapping trait associated genes in admixture populations (Lindtke et al., 2013), correcting for population stratification in genome-wide association studies (Joukhadar et al., 2013), improving breeding programs (Migicovsky et al., 2016), estimation of recombination rate (Wegmann et al., 2011), imputing variants (Paşaniuc et al., 2009), and localizing unmapped sequences on reference genomes (Genovese et al., 2013).

Over the last two centuries, Australia has transformed from a country that imports wheat to one of the world's largest wheat exporters (Henzell, 2007). What changes happened to Australian wheat during this relatively short period? How did those changes affect overall genetic diversity in the Australian wheat germplasm pool? From which global regions did Australia import its cultivars or breeding parents through time? Answering these questions can improve our understanding for how wheat was adapted to Australian climates in order to better plan Australia's national breeding programs and future management to adapt to climate changes. In this paper, we studied the global ancestry of Australian wheat germplasm. We also track the geographical ancestry of the Australian bread wheat germplasm using *in silico* chromosome painting based on SNP markers by comparing it to a worldwide wheat population. We examined the origin of ancestors that dominated the Australian germplasm at any period since 1840 or have dominated different Australian wheat growing regions. We also estimated the changes in genetic diversity through time and calculated the effective population size in different time periods.

## Materials and methods

### Plant materials

**Figure 1:**
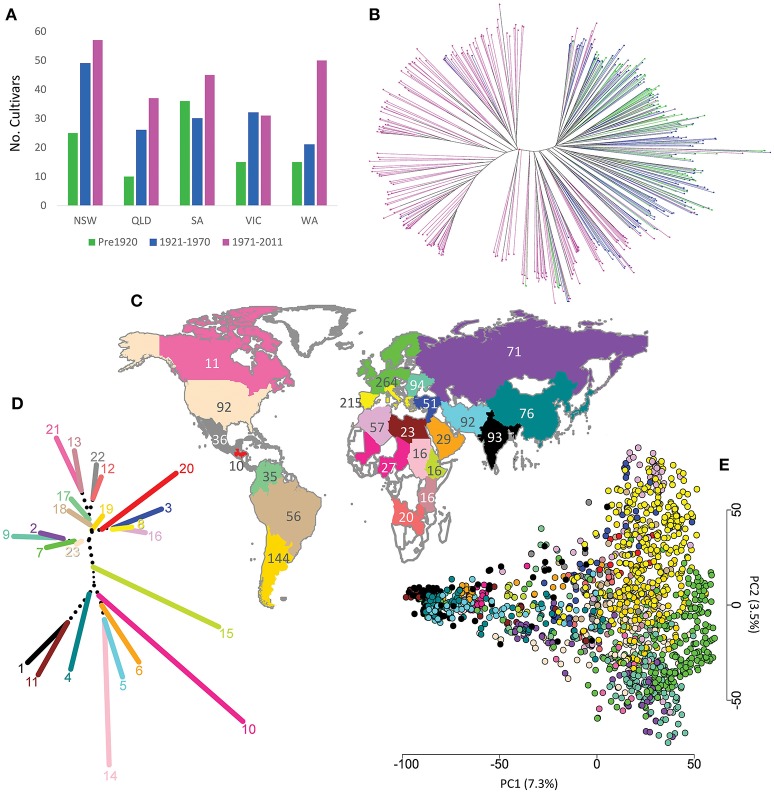
**(A)** Number of Australian cultivars grouped by year and State of release; **(B)** phylogenetic tree for the 482 Australian cultivars, colors describe the cultivar release period as in **(A)**; **(C)** distribution and number of worldwide accessions used in this study; **(D)** Neighbour-Joining clustering of worldwide subpopulation using pairwise *Fst* matrix, colors describe worldwide subpopulations as in (Figure 1C); **(E)** distribution of worldwide accessions based on the first two principal components. NSW, New South Wales; QLD, Queensland; VIC, Victoria; SA, South Australia; WA, Western Australia.

Two bread wheat populations (donor and recipient) were used for this research. The aim is to infer from which ancestral donor population the recipient population originated. The Australian germplasm (Table S1), designated the recipient population, was comprised of 482 cultivars obtained from the Australian Grains Genebank (AGG), Horsham. Released between 1840 and 2011, these cultivars represent the breeding history of bread wheat in Australia (Figure 1A, Table S1). The worldwide collection, hereafter described as the donor population was obtained from the AGG and USDA Small Grains Laboratory, and was divided into 23 subpopulations based on their geographical origin (Figure 1C, Table 1, Table S2). We started with a donor population of 2,335 accessions (data not shown) but reduced this number to 1,544 accessions for the *in-silico* painting analysis as described below. The reduced set can be considered as representative for the geographical subpopulations and has low admixture across geographies. The Australian cultivars were compared to the worldwide collection to define the geographical ancestral background of Australian wheat germplasm.

**Table 1:** Details of the worldwide subpopulations.

**PopID**	**Subpopulation country of origin**	**Continent**	**No. of accessions**	**Color**
1	Bhutan, India, Nepal	Asia	93	
2	Armenia, Azerbaijan, Georgia, Kazakhstan, Kyrgyzstan, Russia, Uzbekistan	Asia	71	
3	Jordan, Lebanon, Palestine, Syria, Turkey	Asia	51	
4	China, Japan, Korea	Asia	76	
5	Afghanistan, Iran, Pakistan	Asia	92	
6	Oman, Saudi Arabia, Yemen	Asia	29	
7	Austria, Belgium, Denmark, Finland, France, Germany, Ireland, Netherlands, Norway, Poland, Sweden, Switzerland, United Kingdom	Europe	264	
8	Greece, Italy, Portugal, Spain	Europe	215	
9	Bulgaria, Moldova, Romania, Ukraine	Europe	94	
10	Chad, Mali, Nigeria	Africa	27	
11	Egypt, Libya	Africa	23	
12	Angola, Zambia, Zimbabwe	Africa	20	
13	Burundi, Kenya, Tanzania	Africa	16	
14	Sudan	Africa	16	
15	Eritrea, Ethiopia	Africa	16	
16	Algeria, Morocco, Tunisia	Africa	57	
17	Colombia, Ecuador, Venezuela	S America	35	
18	Bolivia, Brazil, Paraguay, Peru	S America	56	
19	Argentina, Chile, Uruguay	S America	144	
20	Honduras, Guatemala	N America	10	
21	Canada	N America	11	
22	Mexico	N America	36	
23	United States	N America	92	

### Genotyping and imputation

DNA was extracted from leaf tissue collected at the 2-leaf seedling stage from a single plant per accession. Both the donor and recipient populations were genotyped with the Infinium iSelect 90K SNP bead chip assay described in Wang et al. (2014). GenomeStudio polyploid clustering V1.0 software (Illumina Ltd.,) was used to export normalized NormR and Theta values for each accession for SNPs that produced well-separated clusters for unambiguous scoring and had been previously genetically mapped (Wang et al., 2014). SNP genotype calling was performed using a custom PERL script that assigned a genotype to each accession based on the Euclidian distance of the sample data point to the center of pre-defined clusters having known allelic relationships, considering the standard deviations of the defined clusters. A total of 14,898 polymorphic SNPs were obtained. Following filtering to remove SNPs with >50% missing data, the remaining 13,159 SNPs were imputed using LinkImpute software with default parameters (Money et al., 2015). After imputation, SNPs without position on the consensus map and SNPs with a minor allele frequency (MAF) <5% in the recipient population were removed, leaving 8,212 SNPs. SNPs that were common between both the donor and recipient populations (total of 8,194) were used in subsequent analyses. The Genotype data for Australian and worldwide collection can be downloaded from https://doi.org/10.5061/dryad.06c67

### Statistical analysis

#### Diversity, LD and N_e_ estimation in Australian wheat

Australian wheat germplasm encountered two major changes during its evolution: in 1901, when Farrer introduced earlier maturing cultivars; and in 1970, when semi-dwarf wheat was introduced (Pugsley, 1983). For this reason, the recipient population was divided into three subpopulations based on the cultivar year of release: pre 1920; from 1921 to 1970; and post 1970. We arbitrarily set the end of the first period in our germplasm to 1920 (instead of 1900) because we could obtain only 38 cultivars that were released during the nineteenth century, of which 31 were from NSW and SA. Moreover, crosses between 1901 and 1920 were less dependent on the early maturity cultivars compared to subsequent years (Table S1). State and year of release information was obtained from the AGG and wheat GRIS database (http://wheatpedigree.net/).

To assess the genetic diversity within each time period, we calculated the average haplotype heterozygosity and the average effective number of haplotypes as haplotype-based analyses are reported to better mitigate ascertainment bias compared to single-marker analyses (Conrad et al., 2006). The haplotype heterozygosity was estimated as: $HHe=1-{\sum p}_{i}^{2}$; where *p*_*i*_ is the frequency of the haplotype *i*. The effective number of haplotypes was calculated as: $HHe=1/{\sum p}_{i}^{2}$. The analysis was run with a sliding window of size 15 SNPs that moves one SNP each time. Different window sizes ranging from 15 to 25 were also tested but consistent results were obtained. The window size was defined based on the linkage disequilibrium results and marker coverage (Bonnen et al., 2002; Xu et al., 2009).

The phylogenetic relationship among Australian cultivars was determined by calculating Nei's genetic distance among all cultivars, followed by Neighbour-Joining clustering. *Fst* was calculated among the three subpopulations following Weir and Cockerham (1984) to estimate the differentiation among cultivars released in each time period. To further confirm the differentiation between the three subpopulations, AMOVA was also estimated treating the three time periods as three subpopulations distributed in one or two regions (before 1970 and after 1970) using GenAlex software (Peakall and Smouse, 2006). In other words, for the two region analysis, we considered the first two periods as one region in AMOVA in order to better understand the differentiation of the most recently released cultivars. The effective population size (*N*_*e*_) was estimated for each subpopulation using the linkage disequilibrium method under a random mating assumption implemented in NeEstimator V2 software (Do et al., 2014).

Linkage disequilibrium (LD) was calculated according to Hill and Robertson (1968) as a pairwise *R*^2^ for each chromosome using the R package “*snpstats*” (Clayton, 2015). *R*^2^ values were plotted against the genetic distance between each pair of SNPs in order to compare the decay of LD among Australian cultivars from each time period. In order to determine the critical value for *R*^2^, *R*^2^ was calculated for each pair of SNPs from different chromosomes (unlinked SNPs). The 99th quantile of all unlinked *R*^2^ values was considered as the baseline beyond which genetic linkage is likely to cause LD.

#### Australian wheat global ancestry analyses

A hundred replicates of the *ADMIXTURE* analysis with K between 2 and 20 were run with 10 cross validations to estimate the number of underling populations (Alexander et al., 2009). SNPs were pruned for LD at an *R*^2^ value equal to 0.5 using PLINK as *ADMIXTURE* assumes unlinked variants. However, as the average cross-validation values over the 100 replicates showed a continual decrease with the increase in K, and, therefore, we were not able to determine the most probable K for our germplasm. For this reason, we investigated the convergence of the top 20% replications with the highest Log-Likelihood values at each K (Behar et al., 2010; Pagani et al., 2016). The most probable *K*-values should have identical or similar Log-Likelihoods in those top 20% of replicates. To further validate and compare the subpopulations obtained by *ADMIXTURE*, we ran the discriminant analysis of principal components (DAPC), implemented in the R package “*adegenet*” (Jombart et al., 2010). First, we ran the k-means clustering with values ranged between 2 and 50 and the most probable K was declared using the Bayesian Information Criterion (BIC) which was equal to 12. Second, we validated our population structure model given *K* = 12 using DAPC analysis. The optimal number of PCs to be included in the analysis was defined using the function “*optim.a.score*,” which was 56 PCs.

The linked model implemented in FineStructure V2 was also used to cluster the whole Australian germplasm into subpopulations with recommended settings by the developers (Lawson et al., 2012). Each Australian wheat cultivar was considered as a mosaic of all other Australian cultivars. The number of iterations for burn-in and Markov Chain Monti-Carlo (MCMC) was set to 100,000 each in two independent runs. The number of maximization steps when finding the best state from which the tree is built was set to 100,000.

#### Characterizing of donor populations for *In-Silico* chromosome painting analysis

As indicated above, we started with 2,335 worldwide accessions to define a number of differentiated donor subpopulations for the chromosome painting analysis. We first calculated Nei's genetic distance among all accessions followed by Neighbour-Joining clustering for the whole donor population. Second, we omitted highly admixed clusters that had accessions originated from broad geographical origins. Third, we defined 23 major clusters that involved accessions from neighboring countries. Finally, we removed individual accessions that clustered away from their core subpopulations or accessions that were clustered with different geographical regions. Different filtering steps resulted in a donor population of 1,544 accessions divided into 23 different geographical subpopulations (Table 1).

We assumed that the above-mentioned reduction strategy would increase the differentiation between donor subpopulations and increase power for the *in-silico* chromosome painting analysis. To confirm this assumption, analysis of molecular variance (AMOVA) was performed for both the full set and the subset considering 23 subpopulations (Table 1) and five regions representing the five continents of origin. Pairwise Fixation index (*Fst*) was also calculated among the 23 subpopulations before and after selecting the subset. To visualize subpopulation relationships, a Neighbour-Joining phylogenetic tree for the 23 reduced subpopulations was plotted using the *Fst* matrix. The principal component analysis (PCA) was calculated using the R function “*prcomp*” and the first two principals were used to plot the distribution of the reduced worldwide population of 1,544 accessions.

To confirm that *Fst* increase after the reduction was not just a result of reducing the population size, we ran 100 replicates of a simulated reduced set with 1,544 random accessions selected from the original 2,335 accessions. In those 100 replications, each subpopulation received a number of random individuals equal to that in the reduced set and *Fst* values were averaged among all subpopulations. The mean and standard deviation were calculated for those 100 average *Fst* values in order to make statistical inferential about our reduced set average *Fst* value.

#### *In silico* chromosome painting of Australian wheat

To infer the local ancestry of the Australian wheat germplasm, we used the algorithm implemented in ChromoPainter software (Lawson et al., 2012). This method tests the admixture of populations by considering the recipient chromosome (Australian germplasm) as a mosaic of the donor chromosomes (worldwide germplasm). First, the method uses a user defined number of steps (or iterations) for Expectation Maximization algorithm (in our case we used 20 steps) to estimate the probability of copying from each donor. Results of each step are considered as the prior probability for the following Expectation Maximization step. The ChromoPainter algorithm then uses the final step probabilities in a modified approach of the Hidden Markov Model proposed by Li and Stephens (2003) to paint recipient chromosomes as a series of regions obtained from the donor chromosomes. It considers the allelic state of donor and recipient SNPs, as well as SNP linkage along the chromosomes to increase the detection power. One hundred replicates were run for each recipient individual and the most frequent donor population in these 100 replicates at each SNP was considered as the ancestral donor for the SNP. The first analysis used all 23 donor subpopulations. In a subsequent repeated analysis, we omitted African subpopulations 11, 12, and 16 (Table 1). As we describe in the Discussion, those three African subpopulations showed a large contribution to the Australian germplasm that was inconsistent with Australian historical records. For this reason, removing them allowed for cleaner chromosome painting (Hellenthal et al., 2014) and a better understanding of the evolution of the Australian germplasm.

### Gene identification

Unique genomic regions in recipient accessions released after 1970 were first defined using the 90K SNP consensus genetic map (Wang et al., 2014). Probe sequences of SNPs in these regions were aligned against the IWGSC genome assembly v1.0 of wheat cultivar Chinese Spring (https://www.wheatgenome.org) using GYDLE software (https://www.gydle.com/) to determine their physical positions and to extract flanking sequences. Annotated wheat genes and their associated gene ontology (GO) terms were downloaded using the Biomart tool available at the Ensembl website (http://plants.ensembl.org/biomart/martview) and those genes were aligned against the extracted regions of interest using BLAST+ software (Camacho et al., 2009). Aligned genes with over 99% identity and at least 70% coverage were assigned to those regions.

## Results

### Australian wheat diversity

To examine the evolutionary history of Australian wheat germplasm, representative cultivars from 1840 to 2011 were divided into three periods separated by two time points (1920 and 1970). Very low differentiation among cultivars released in the first two time periods was observed (*Fst* = 0.01). In contrast those released after 1970 were more differentiated, having *Fst*-values of 0.14 and 0.12 with cultivars released pre and post 1920 respectively. These findings were similar to the phylogenetic analysis (Figure 1B) and the AMOVA results which considered the first two time periods as one region and the third period as a second region. The AMOVA revealed that only 1% of the total variation could be attributed to variation among subpopulations and 12% for variation between regions (Table S3A). When considering the three periods as one region, 11% of the total variation was attributed to variation among the three subpopulations (Table S3B). Further, the allele frequency for SNPs across the genome was very similar for the first two periods (pre-1920 and 1921–1970) compared to the third one (Figure S1). For example, Figure S1 shows a clear difference in the frequency of the SNPs that flank the Green Revolution genes *Rht-B1* and *Rht-D1* in the third period, compared to the first two periods.

**Figure 2:**
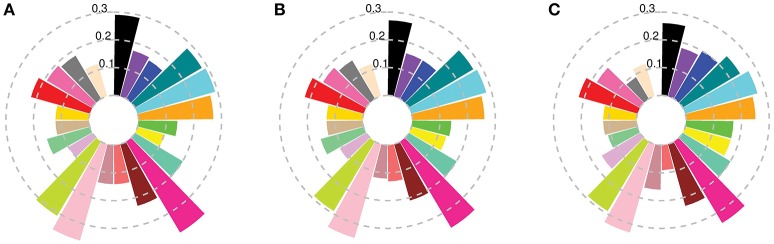
*Fst* between worldwide subpopulations and Australian cultivars released **(A)** before 1920; **(B)** from 1921 to 1970; and **(C)** after 1970. Colors describe worldwide subpopulations following Figure 1C.

When compared to the worldwide germplasm, subpopulations 8 and 16 were the closest to the cultivars released in the first two time periods, while subpopulations 12 and 22 had the lowest differentiation from the most recent (1971–2011) cultivars (Figure 2, Table S4A). Dividing the Australian germplasm based on the State in which they were released did not convey any additional information or special features associated with the different Australian States, except that Queensland had a slightly higher differentiation from the other States, with a maximum *Fst* value of 0.08 with South Australia (Table S4B). AMOVA analysis indicated that only 4% of the total variation resulted from variation between States (Table S3C).

**Figure 3:**
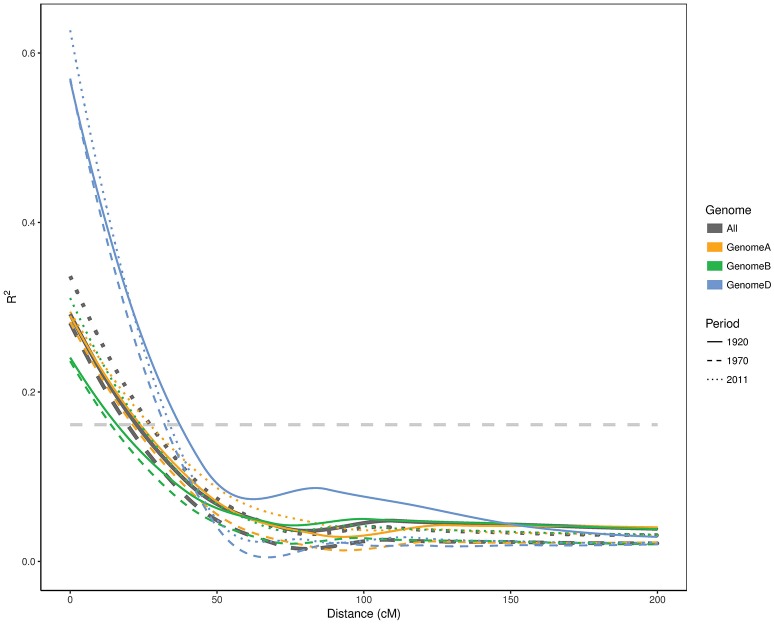
Linkage disequilibrium decay in **A**, **B**, and **D** genomes as well as whole genome of Australian cultivars released in different time periods. The horizontal line represents the LD threshold.

Although the first two time periods seem to be very similar, the effective population sizes (*N*_*e*_) varied substantially. *N*_*e*_ was 11.5 for the first period (pre-1920) and increased to 23 in the second period (1921–1970), despite the population size being only 1.5 fold larger. The population size for the last period cultivars (1971–2011) was 2.2 times larger than the first period (pre-1920) subpopulation, yet it had the lowest *N*_*e*_ value (9.4). To further confirm these changes in genetic diversity in the Australian germplasm, we estimated the LD decay for each genome in each time period (Figure 3). The background LD was equal to 0.161 which is the threshold to declare linkage as causal for LD. The second time period had the smallest (19.4 cM) LD blocks followed by the first and third periods with 22.5 and 27.2 cM, respectively. Given that we had one SNP every ~1.32 cM and the second time period had the smallest LD blocks with 19.4 cM (Figure 3), we estimated that 15 SNPs was a good approximation for the window size in the haplotype heterozygosity (*HHe*) and the effective number of haplotypes (*HAe*) analyses. *HHe* confirmed that the second period had the highest diversity with an average *HHe* across all haplotypes of 0.840, followed by the first period with *HHe* = 0.829 and then the third period with *HHe* value of 0.827. Similarly, *HAe* values of 9.0, 10.2, and 8.8 were obtained for the first, second and third periods, respectively.

### Global ancestry of Australian wheat

**Figure 4:**
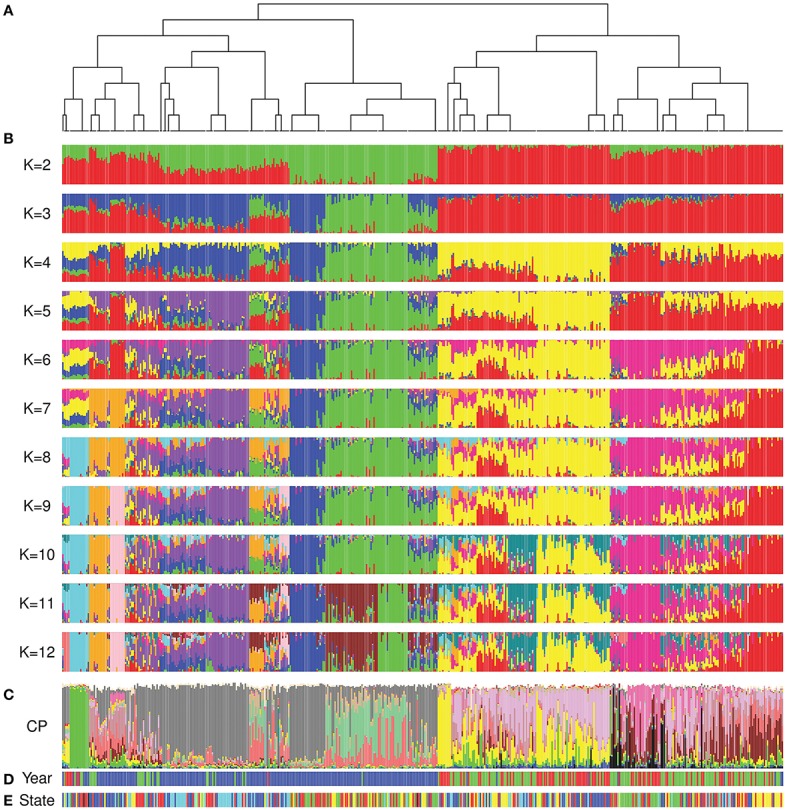
Comparison of FineStructure, *ADMIXTURE* and ChromoPainter results. In all analyses, Australian cultivars are sorted by the order that they appear in the FineStructure dendrogram. **(A)** the dendrogram of the 38 subpopulations revealed by FineStructure; **(B)** *ADMIXTURE* analysis (K range from 2 to 12) for the whole Australian germplasm; **(C)** ChromoPainter results for the whole Australian germplasm (proportion of ancestral contribution of each of the 23 donor subpopulations for each Australian cultivar). Colors describe worldwide ancestral subpopulations following Figure 1C; **(D)** Colours represent accession year of release (red: pre-1920, green: 1921 to 1970, and blue: post-1970); **(E)** Colors represent the State of release (red: NSW, green: QLD, blue: SA, yellow: VIC, and cyan: WA).

The FineStructure and *ADMIXTURE* analyses showed comparable results and confirmed the differentiation of cultivars released after 1970 (Figures 4A,B; Figures S2, S3). As previously stated, we were not able to estimate the number of underlying populations using a cross-validation approach. For this reason, we investigated the convergence of the highest 20% Log-Likelihood values at each K following Behar et al. (2010) to suggest the most probable K value(s). The most probable K with this method ranged from 2 to 9 as well as 12. The k-means analysis showed that 12 was the most probable K to describe our population (Figure S4A) while the DAPC scatterplot showed a clear separation of the 12 groups obtained by *ADMIXTURE* analysis (Figure S4B). Moreover, the average Fst value for the 12 subgroups was equal to 0.52 indicating that they have high differentiation. The FineStructure analysis revealed 38 subpopulations (Table S1) clustered in two major clades, before 1970 and after 1970 with minor overlaps. Figure 4A shows the FineStructure clustering of the 38 populations and the *ADMIXTURE* results (Figure 4B) for those populations with K ranging from 2 to 12. Like previous analyses, cultivars released in different States (Figure 4E) did not appear to be highly structured or isolated from each other, although some were more structured than others such as Queensland (QLD) before 1970 and Western Australia (WA) after 1970 (Figure 4D,E; Figure S2). Moreover, the overall look at the ancestry proportion for each State indicated some variation among different States (Figure S5).

### Diversity of the donor populations

The full worldwide wheat population set of 2,335 accessions showed moderate gene flow between different subpopulations with an average *Fst* value among subpopulations of 0.132 (data not shown). AMOVA analysis revealed the highest proportion of total variation (89%) was amongst individuals within subpopulations, followed by 8% for variation amongst subpopulations and 3% for variation amongst continents (Table S3D) which is comparable to previous reports in hexaploid wheat (Manickavelu et al., 2014; Nielsen et al., 2014). By removing mixed phylogentic clusters and reducing the worldwide population size to 1,544 the average *Fst* value among subpopulations increased to 0.237 (Table S4). Running 100 simulated reduced sets resulted in average *Fst* value amongst subpopulations of 0.129 ± 0.007 indicating that this increase in *Fst* values was not a result of reducing the population size (*p*-value ≈ 0). The lowest *Fst* (0.05) was between South European (Greece, Italy, Portugal, Spain) and North African countries (Morocco, Algeria, and Tunisia), while the highest value (0.55) was between Canada and central African countries (Chad, Mali and Nigeria). AMOVA analysis of the reduced worldwide population set partitioned the total variation as 16% amongst subpopulations and 81% within subpopulations (Table S3E). Figure 1D shows Neighbour-Joining clustering of the reduced 23 worldwide subpopulations using the pairwise *Fst* matrix, and Figure 1E shows the distribution of the worldwide accessions using the first two principal components. This analysis clustered the accessions of the same population close to each other with some overlapping among different subpopulations. Given that the 23 subpopulations showed good differentiation based on their *Fst* values (Table S4), lack of clustering for some subpopulations in the PCA can be explained by the low explanation of the first two PCs (only 10.8%) of the total variation in the germplasm.

### *In silico* chromosome painting of Australian wheat

**Figure 5:**
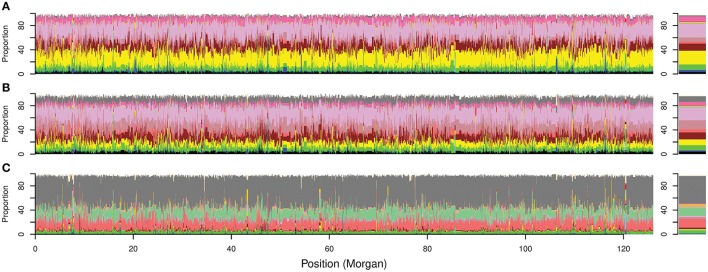
Overall local ancestry results along Australian bread wheat genome. Proportion of ancestral contribution for each donor subpopulation at each SNP locus across the genome. The 21 chromosomes are ordered sequentially starting from chromosome 1A to 7D for cultivars released **(A)** before 1920; **(B)** from 1921 to 1970; and **(C)** after 1970. Right bars represent the overall contribution for each donor subpopulation to the ancestral makeup of the Australian germplasm. Colors describe worldwide subpopulations following Figure 1C.

*In silico* painting of the chromosomes of the Australian cultivars as recipients of DNA segments originating from worldwide donors revealed a complex mosaic structure in the Australian germplasm (Figure 4C; Figure 5, Figure S6). Before 1970 ancestral origins were more diverse compared to post 1970 cultivars. A detailed examination of the ancestral origins of inherited DNA across the genome in each time period revealed that wheat with South European origin (subpopulation 8) contributed 22.6% to the ancestral makeup of cultivars from the first time period, followed by North African origin, with subpopulations 16 and 11 contributing 22 and 11.5%, respectively (Figure 5). During the second time period, African materials became more important with subpopulations 16, 13, and 11 contributing 21, 14.9, and 11.3% of inherited DNA in the Australian germplasm, respectively. However, Southern European origin continued to be an important ancestor during this period with 9.1% contribution. The results were comparable to the FineStructure results which divided pre-1970 germplasm into two main clusters (Figure 4), of which the first contained cultivars painted to South European and North African countries (~48% of this cluster from the first period), while the second contained cultivars painted mainly to multiple African subpopulations (~33% of this cluster from the first period).

In contrast to the first two periods, the ancestral makeup of cultivars from the third time period depended mainly on Mexican materials, which contributed 45% of the inherited DNA, followed by central African subpopulation number 12 with 14.6% and the northern subpopulation in South America (subpopulation 17) with 13% (Figure 5). Comparing the *ADMIXTURE* and FineStructure results (Figure 4; Figure S2), cultivars that were almost fully painted to Mexico were clustered in two main subpopulations (blue and purple in *ADMIXTURE K* = 12). The blue subpopulation involved cultivars released in New South Wales (NSW) and QLD, while the purple subpopulation involved cultivars released in South Australian (SA), WA and NSW (especially the materials with Gabo background released before 1970 Figure 4, Figure S2).

**Figure 6:**
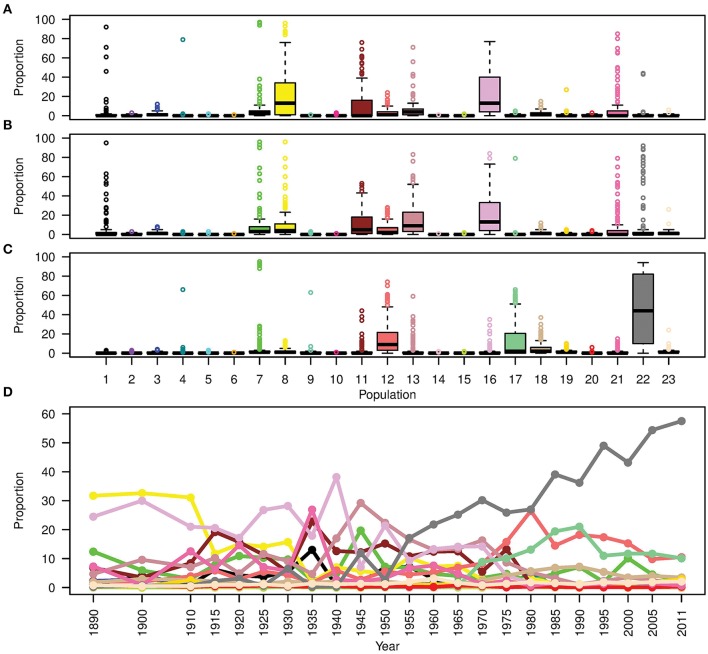
Boxplots showing the contribution (expressed as a proportion) of each donor subpopulation on all recipient cultivars released **(A)** before 1920; **(B)** from 1921 to 1970; and **(C)** after 1970. **(D)** The contribution of donor subpopulations to the Australian germplasm through time. Colors describe worldwide subpopulations following Figure 1C.

To show the evolution of Australian germplasm through time in more detail, Figure 6D shows the ancestral makeup of the Australian germplasm across a finer time scale. This figure clearly demonstrates a dependency on subpopulations 8 and 16 during the pre-1920 period, the complex constitution during the second period (1921–1970) and the gradual increase in use of Mexican materials post 1971 to reach a peak at 57.5% in 2011. Considering the effect of each donor subpopulation on individual Australian cultivars (Figures 6A–C), it was clear that the contribution of each donor subpopulation to the composition of the germplasm in each time period was variable. For example, the contribution of subpopulation 22 in the post 1970 cultivars showed a large interquartile range (Figure 6C), while minor donor subpopulations contributed a large proportion of some individuals; e.g., the outliers in subpopulations 1, 4, 7, 17, 21, and 22 for the first two periods and subpopulations 4, 7, 9 13, and 18 for the last period (Figures 6A,B).

Analysis of the proportion of each ancestral subpopulation for cultivars released in each State in each period (Figures S6, S7) showed that the similarity to Mexican materials started earlier in NSW than in other States (purple subpopulation in the *ADMIXTURE* analysis *K* = 12, Figure 4). The dependency on subpopulation 17 after the green revolution was minimal in SA and WA when compared to the other States (Figure S7). The analysis also confirmed the differences in ancestral makeup of cultivars released in QLD before 1970 and their low dependency on European ancestors.

Interestingly, after 1970, chromosome 1B showed the lowest dependency on Mexican wheat which contributed only 39.5% of the chromosome 1B ancestral makeup. This can be explained by strong selection in Australian breeding programs against the 1B/1R translocation, which carried resistance to four major wheat diseases: powdery mildew, stripe rust, leaf rust and stem rust but can reduce grain quality (Dhaliwal et al., 1987). This resulted in the chromosomal region harboring the 1B/1R translocation having only 34.5% Mexican ancestral origin (Figure S8). Cultivars released after 1970 had numerous small regions distributed across the genome that deviated from the Mexican germplasm (Table 2). These regions and their ancestral subpopulations are summarized in Table 2. Those regions underlined genes with mainly binding, catalytic, transferase, hydrolase and kinase activities which are involved in metabolic, biosynthetic and response to stress processes (Table S5).

**Table 2:** Genomic regions that have major ancestors other than Mexican wheat for cultivars released after 1970.

**Region Name**	**Chr**	**Region Start**	**Region End**	**Dominant Subpopulations**
chr1A_1	1A	566075013	572828305	12,22
chr1B_1	1B	1252541	4692345	11,19,22
chr1D_1	1D	484409375	495229051	7,8,17,22
chr2A_1	2A	185122041	210470077	12,22
chr2B_1	2B	730563021	734200089	12,22
chr2D_1	2D	635950569	650322707	10,12,22
chr3A_1	3A	641407271	654318730	17,21,22
chr3B_1	3B	5488617	12671303	18,19,22
chr3B_2	3B	93789843	139767779	12,22
chr3B_3	3B	64287854	81567861	19,22
chr3B_4	3B	542463890	580968645	11,17,22
chr3B_5	3B	591329634	647248232	17,22
chr3B_6	3B	750588195	752880689	4,12,22
chr4B_1	4B	595709044	604148303	12,22
chr5A_1	5A	31601578	126693184	8,12
chr5A_2	5A	461484554	480041044	12,17,22
chr5B_1	5B	7706162	8945959	18,22
chr5B_2	5B	232214886	244411624	7,22
chr6A_1	6A	18464439	23439107	18,22
chr6A_2	6A	381025560	447898193	17,22
chr6B_1	6B	167152	10744763	8,12
chr6B_2	6B	390044201	439065431	7,12,22
chr6B_3	6B	703390755	711756871	12,15,22
chr6D_1	6D	3726727	5725474	13,17,22
chr7A_1	7A	14648701	19896551	12,22
chr7A_2	7A	115935384	149233471	7,12,18,22
chr7A_3	7A	644044436	686723088	12,22
chr7A_4	7A	708291263	717184159	8,12,22
chr7B_1	7B	115785950	136288734	19
chr7B_2	7B	584281004	588227382	18,22
chr7B_3	7B	733490273	749551593	5,7

*Genomic positions are reported relative to IWGSC genome assembly v1.0 for cultivar Chinese Spring (http://www.wheatgenome.org)*.

The ancestry results revealed by *in silico* painting showed good concordance with pedigree records (Table S1). A number of cultivars selected or derived from Purple Straw that dominated Australian wheat production approximately between 1890 and 1920 were traced to south European countries that included Italy, the origin of Purple Straw (Figure S9A). The Canadian cultivar Red Fife was used in breeding during the early years of the twentieth century (Table S1), and a number of its decedents were painted to Canada (Figure S9B). Similarly, between 1938 and 1969, a number of Kenyan accessions were used (Table S1) and their descendants showed clear Kenyan ancestry (Figure S9C). Gabo, a cultivar bred in Australia and used extensively in the CIMMYT wheat breeding program, was painted to Mexico (Figure S9D), mainly to the purple subpopulation in the *ADMIXTURE* analysis (Figure 4). Cultivars derived from CIMMYT such as cultivar WW15, which involved a triple backcross with cultivar Andes-Enano from South America, were painted to subpopulation 17 which included the Andes region in South America (Figure S9E).

To gain a better understanding on the evolution of Australian wheat and obtain cleaner chromosome painting results, we repeated the analysis after excluding donor subpopulations 11, 12, and 16 which showed an unexpectedly large contribution to the Australian wheat ancestral makeup (Figures S10, S11). Following pedigree records, these subpopulations are unlikely to contribute largely to the Australian wheat germplasm and their contribution may be just a result of gene flow among donor subpopulations. After removing those subpopulations, south European countries formed 40.3% of the germplasm in the first time period and 23.2% in the second period, while Kenyan subpopulations contributed 14.1 and 25.7% to Australian germplasm released in the first and second periods, respectively. Interestingly, even after the cleaned painting clearly showed a European contribution to the Australian germplasm, QLD continued to show very low admixture with European subpopulations (Figure S10). Regarding the third period (post 1970), 51.2% of the germplasm was attributed to the Mexican subpopulation. During the first two periods, Indian and Canadian materials made a larger contribution to the Australian ancestral makeup with 8.1 and 12.4% during the first period and 9.3 and 7.9% during the second period, respectively. In the last period, South American subpopulations (subpopulation 17 and 18) had higher contribution to Australian germplasm with 16.6 and 8.1%, respectively (Figure S11).

## Discussion

When the First Fleet arrived in Australia in 1788, they brought British bread wheat cultivars with them because neither hexaploid wheat nor any of its relatives existed in the new land. Later, they imported cultivars from other regions because Australia's dry climate was very different to that of the UK (Henzell, 2007). For this reason, the evolution of Australian wheat can be characterized as two centuries of extensive selection and hybridization on wheat originally adapted to other geographical regions, rather than the domestication of wheat from locally adapted wild relatives. The Australian breeding effort involved wheat materials from broad origins, as was recognized by the famous Russian botanist and geneticist Nikolai Vavilov in 1935 when he acknowledged Farrer's breeding program: “*There is probably no region where intraspecific and interspecific hybridization of wheat has been so extensive as in Australia*” (Atwell et al., 1999). While the general theme of Australian germplasm has been recorded in cultivar pedigree information, it is only now that detailed information describing each cultivar's geographical origins at the genomic level can be resolved using advanced molecular and statistical tools. Here we have shown that understanding the population structure and ancestral genomic composition of Australian wheat can clarify the evolution of the germplasm pool, and facilitate planning for future breeding programs by allowing for better management of available genetic variation.

### Effect of donor and recipient populations on the chromosome painting analysis

The algorithm implemented in ChromoPainter (Lawson et al., 2012) allows the accurate tracking of whole genome local ancestry for each recipient individual by comparing it to a worldwide collection. This method detects the most recent common ancestor for each recipient locus in a set of donor subpopulations even when the actual donor subpopulations has high differentiation from the recipients. For example, subpopulation 11 had *Fst* > 0.2 with the Australian subpopulations from the first two time periods (Figure 2) but it constituted around 11.4% of their total ancestral makeup (Figures 5A,B). The first two time periods had very low (0.01) differentiation, but showed considerably different ancestral makeup (Figures 5A,B). The painting results were generally consistent with pedigree records (Figure S9; Table S1), reflecting the accuracy of the method.

The history of wheat in Australia is relatively short: about 228 years, of which approximately 177 years corresponds to the breeding of our germplasm. In contrast, the domestication of bread wheat started about 10,000 years ago (Dubcovsky and Dvorak, 2007). For this reason, the time to the most recent common ancestor for any Australian wheat cultivar is relatively short, which increases the power of ancestry detection (van Dorp et al., 2015). Painting newly evolved populations has the additional advantage of requiring only low SNP density due to the small number of generations separating the recipients from their ancestral populations. The short time separating recipient individuals from their most recent common ancestors can result in large regions of shared DNA (McTavish and Hillis, 2014) because of limited recombination since diversion.

Extensive recent admixture among donor subpopulations can lead to pseudo geographical ancestral relations with recipients. This can be explained by the presence of shared ancestry for the pseudo donor ancestor and recipients, and the short diversion time separating both populations from their common donor ancestor. To accurately detect the true ancestral origin of new populations (such as Australian wheat) and not pseudo ancestors, donor subpopulations need to be carefully selected to represent their geographical origin by (1) minimizing donor subpopulation admixture; and (2) removing individuals that have identical by descent relations with the recipient population. For this reason, we first removed highly admixed phylogenetic clusters in the donor subpopulations that resulted in a doubling of the average *Fst* value. And second, we removed donor individuals that are known to have Australian background such as the Tunisian cultivar Cailloux, which is derived from the Australian cultivar Florence. However, Mexican materials that had Gabo background were not removed for two reasons: CIMMYT's breeding program largely depended on Gabo after its release in NSW, and consequently the removal of these cultivar would have resulted in a very small Mexican donor subpopulation; and second, several regions of the world have adopted CIMMYT cultivars since the Green Revolution (Lantican et al., 2016). The latter can result in misleading conclusions regarding Gabo-derived Australian cultivars. Thus, cultivars with Gabo parents are expected to be painted to Mexico in our analysis. Another important issue is that even after doubling the average *Fst* value between donor subpopulations, we observed some pseudo ancestral relations, as we will discuss later. For those cases, we applied cleaner painting by removing affected donors to gain a better understanding about recipient ancestry (Hellenthal et al., 2014).

### The evolution of Australian wheat germplasm

The beginning of the twentieth century marked a new era for Australian wheat breeding due to the use of early maturity cultivars, such as Federation, that could better tolerate Australian conditions (Wenholz, 1930; Pugsley, 1983). Macindoe and Walkden Brown (1968) as well as (Henzell, 2007) traced the origin of Purple Straw to the British cultivar Red Straw while others declared it was selected from the Italian landrace Tuscan (Quirk, 1982; Marino, 2012). In our analysis, Purple Straw derived varieties were painted to Southern Europe supporting the Italian origin. Surprisingly, Federation and most of its derivatives were mainly painted to the Egypt and Libya subpopulation (subpopulation 11). Applying cleaner painting, which removed Egyptian and Libyan wheat, attributed Federation and its derivatives mainly to India and Kenya. Egyptian and Indian wheat showed the lowest differentiation when the worldwide subpopulations were compared to one another (Table S4). Further pedigree records show that Egyptian germplasm has in general involved crosses with many Indian materials (http://www.wheatpedigree.net; Basnet et al., 2011). Federation-derived cultivars were clustered together in both the *ADMIXTURE* and FineStructure analysis. Interestingly, the Federation genome was exclusively painted to subpopulation 11 while its pedigree also had contributions from Purple Straw and Fife. It seems there might have been unrecorded adoption of Federation derived cultivars in subpopulation 11 since its release in 1901, followed by gene flow to other African regions. Clarification of this finding would require a larger Egyptian and Libyan wheat subpopulation and dating of their admixture with comparison to Australian wheat.

Early generations of Australian wheat breeders crossed or repeatedly backcrossed their cultivars with Purple Straw and other adapted European cultivars (Table S1; Lupton, 1987) and this can explain the low differentiation between cultivars released during the first two time periods. However, extensive artificial selection during the second time period and the introduction of different Kenyan cultivars (Table S1) lead to a shift in the Australian germplasm toward more African-like germplasm during the 1921–1970 period. This occurred as Australia has the same or a similar mega-environment to many African regions (Rajaram et al., 1993) and because of massive gene flow between South European and the North African countries Tunisia, Morocco and Algeria (subpopulation 16). Applying cleaner painting by removing subpopulation 16 increased the South European contribution in the pre-1920 cultivars but increased Kenyan and South European contribution to Australian cultivars from the 1921–1970 period (Figure 5; Figure S11) which supports our conclusion.

Since the beginning of the Green Revolution, Australian wheat breeding is reported to have relied heavily on CIMMYT semi-dwarf materials (Brennan, 1989; Brennan and Quade, 2006). During this period, Australian germplasm significantly changed (Paull et al., 1998; Parker et al., 2002) and the frequency of semi-dwarf genes had increased (Eagles et al., 2009). Our analyses support these records. FineStructure and *ADMIXTURE* clearly differentiated the newer post-green revolution cultivars from older cultivars (Figure 4, Figures S2, S3). *In-silico* chromosome painting also supported this finding, with Mexico attributing 45% of the post 1971 period germplasm in Australia; another 13% was attributed to subpopulation 17 that include Colombia, Ecuador and Venezuela. CIMMYT cultivar WW15 was adopted in Australia and broadly used for breeding. The WW15 pedigree contains a triple backcross with cultivar Andes-Enano that originated from the Andes mountain ranges in South America. This explains the relationship with subpopulation 17. Review of the overall ancestral make up in each Australian State in each period (Figure S7) showed that WW15 derived cultivars were not suited for SA and WA growing conditions, when compared to the other States, an observation previously inferred by pedigree analysis (Brennan and Fox, 1998).

Australian cultivars that were almost fully painted to Mexico were clustered with *ADMIXTURE* and FineStructure in two clades. Each clade contained cultivars released in different Australian States indicating the broad adaptiveness of CIMMYT germplasm across Australia's agri-production zones (Figure 4; Figure S2). Similarly, South Australian cultivars that were painted mainly to subpopulations 12 and 17 showed structure different from cultivars with similar ancestral make up that were released in other States. While *in-silico* chromosome painting could define the ancestral geographical subpopulation of Australian wheat, *ADMIXTURE* and FineStructure analyses further differentiated cultivars painted to the same geographical regions, indicating the complementarity of both strategies.

Unexpectedly subpopulation 12 that includes Angola, Zambia and Zimbabwe contributed 14.6% to the post 1970 germplasm. No historical records were found to demonstrate any relation between subpopulation 12 and Australian wheat. Applying cleaner chromosome painting increased subpopulations 17 and 18 and the Mexican contribution with 3.6, 3.4, and 6.7%; respectively. Tracing of the pedigrees of the subpopulation 12 cultivars revealed a recent dependence on Mexican and Brazilian wheat that can explain this pseudo ancestral relationship with Australian wheat. It seems that both subpopulation 12 and the Australian wheat germplasm have recently adopted similar materials from Mexico and South America that make it hard to specify the exact ancestor without cleaner painting.

During the evolution of Australian wheat, early generation wheat breeders were dependent on wheat from broad geographical origins as a base for their breeding programs. After several generations of extensive breeding, they selected germplasm adapted to Australian climatic conditions that had high genetic diversity compared to post-Green Revolution germplasm. The Green Revolution significantly improved Australian wheat production but resulted in a narrowing of the genetic base and effective population size of the germplasm due to the extensive use of materials with related backgrounds for controlled crosses and artificial selection. The success of semi-dwarf materials shifted the Australian germplasm away from the pre-Green Revolution wheat grown in Australia, as shown by our analysis which revealed differences in both genetic structure as well as geographical origins (Figure 4). This shrinkage in diversity creates a need for urgent actions to cope with future environmental changes. New allelic diversity can be introduced to current Australian germplasm from pre-Green Revolution cultivars or from the geographical regions that dominated the Australian germplasm during the second period such as African and South American countries. Many of these geographical regions have similar climates to Australia and could potentially improve Australian wheat and avoid further loss of genetic diversity.

## Conclusions and future perspectives

Starting from a few poorly adapted British wheat cultivars in 1788 grown in small fields in Sydney, Australia is now one of the largest wheat exporters worldwide. This shift is a consequence of enormous efforts in selecting and hybridizing from cultivars that were originally adapted to other geographical regions. These external resources left DNA landmarks in the Australian wheat genome that have allowed us to understand its history and evolution. With the success of Farrer's breeding program, Australia moved into a new generation of wheat breeding. Australian wheat germplasm became more African-like during the second period and had higher diversity than any other time period. This diversity rapidly shrank with the beginning of the Green Revolution, although grain yield showed significant increases. To maintain diversity and genetic gain into the future, adopting new resources from regions with comparable climates might be required. Mining mid-age Australian cultivars and reutilizing them could be another worthy option as they already have good adaptation to Australian climates, despite having faded from modern Australian wheat. This study highlights the value in tracking ancestry for future breeding activities and faster improvement of yield gain.

## Author contributions

MH and RJ Designed the research; MH provided research materials, analysis tools and worldwide data; RJ performed the research, analyzed the data and drafted the manuscript; MH, HD, and AG supervised the research; MH, HD, AG, and UB. provided substantial comments toward improving the content of the manuscript. All authors read and approved the final copy of the manuscript.

### Conflict of interest statement

The authors declare that the research was conducted in the absence of any commercial or financial relationships that could be construed as a potential conflict of interest.

## Abbreviations

AMOVA: Analysis of molecular variance
AGG: Australian Grains Genebank
CIMMYT: International Maize and Wheat Improvement Center
Fst: Fixation Index
LD: Linkage Disequilibrium
MCMC: Markov Chain Monte Carlo
*N_e_*: Effective Population Size
NSW: New South Wales
QLD: Queensland
VIC: Victoria
SA: South Australia
WA: Western Australia.
