# Supplementary material for: Genetic Diversity, Population Structure and Ancestral Origin of Australian Wheat
Source: Front Plant Sci. 2017 Dec 12;8:2115. doi: 10.3389/fpls.2017.02115 (PMC5733070; doi:10.3389/fpls.2017.02115)
Supplement: Supplementary file 1 [file Image1.PDF]

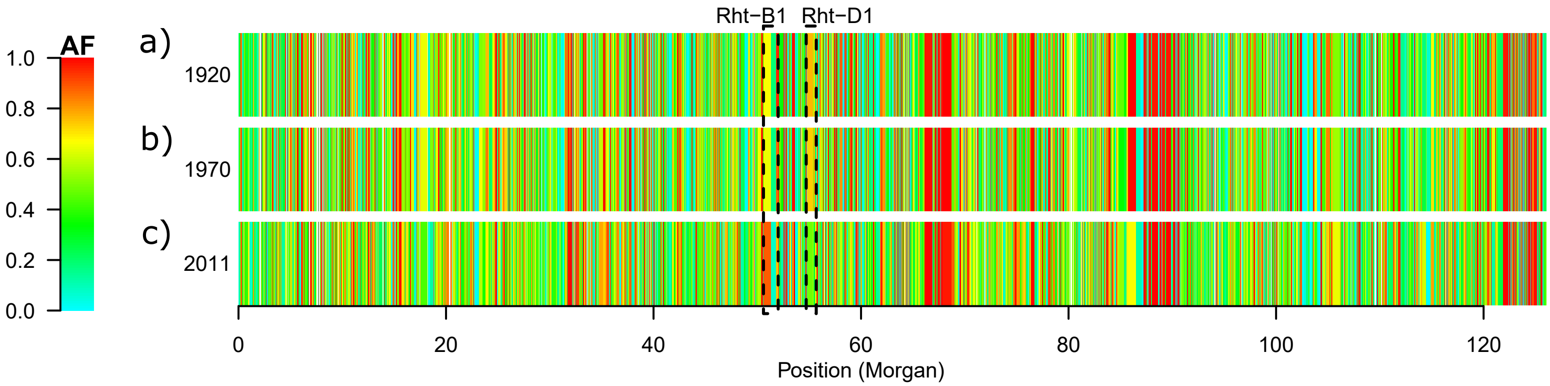

Figure S1. SNP allele frequency along the genome. Each bar represents one SNP and the 21 chromosomes are ordered sequentially starting from chromosome 1A to 7D for cultivars released a) before 1920; b) from 1921 to 1970; and c) after 1970.
