# Supplementary material for: Genetic Diversity, Population Structure and Ancestral Origin of Australian Wheat
Source: Front Plant Sci. 2017 Dec 12;8:2115. doi: 10.3389/fpls.2017.02115 (PMC5733070; doi:10.3389/fpls.2017.02115)
Supplement: Supplementary file 2 [file Image2.PDF]

Figure S2. *ADMIXTURE* analysis (K range from 2 to 12) and *in-silico* painting results for the whole Australian germplasm sorted by state and year of release.

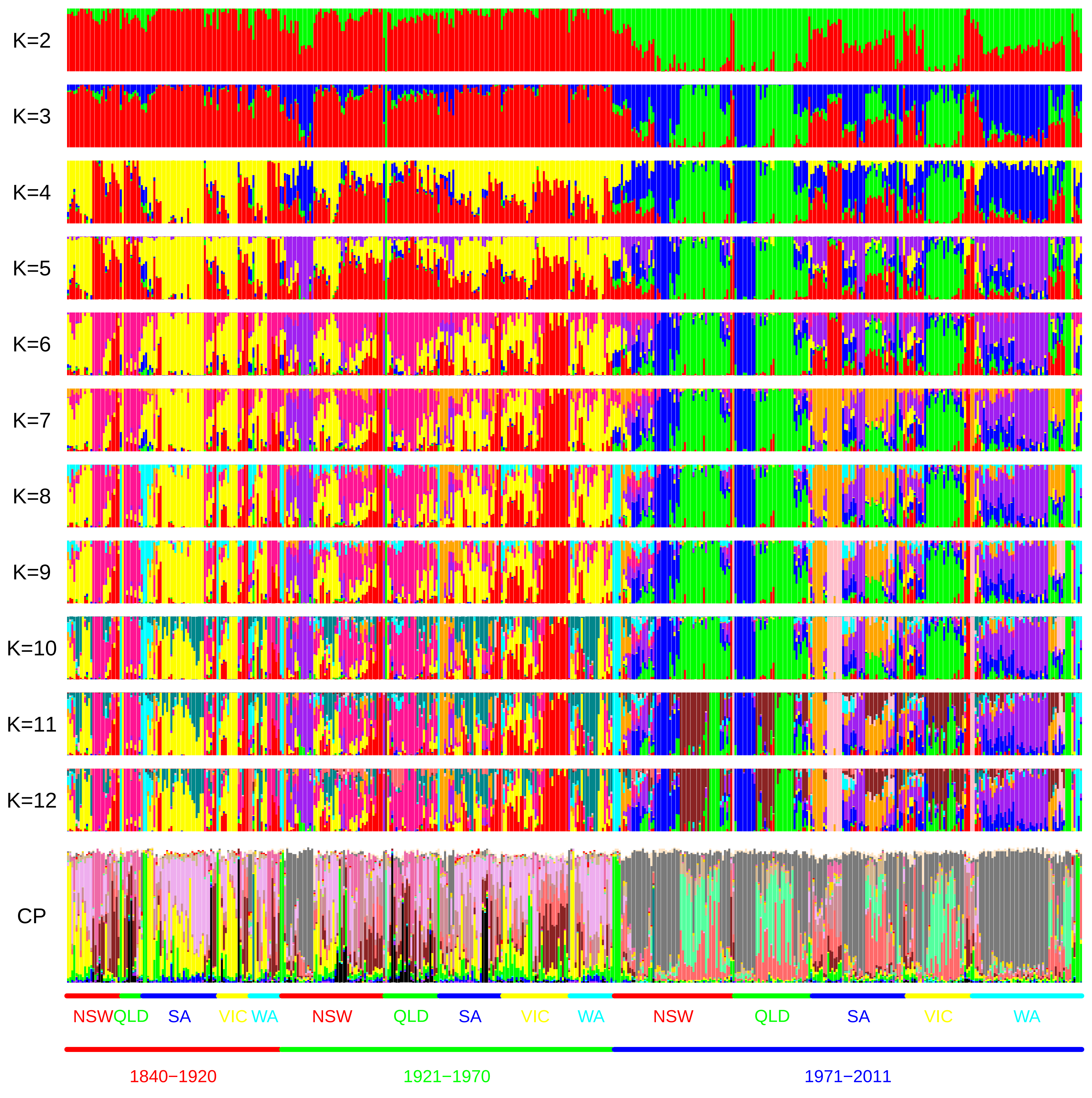

K=2
K=3
K=4
K=5
K=6
K=7
K=8
K=9
K=10
K=11
K=12
CP
NSW QLD SA VIC WA
NSW QLD SA VIC WA
NSW QLD SA VIC WA
1840−1920
1921−1970
1971−2011
