# Supplementary material for: Genetic Diversity, Population Structure and Ancestral Origin of Australian Wheat
Source: Front Plant Sci. 2017 Dec 12;8:2115. doi: 10.3389/fpls.2017.02115 (PMC5733070; doi:10.3389/fpls.2017.02115)
Supplement: Supplementary file 3 [file Image3.PDF]

Figure S3. FineStructure analysis which divided the Australian germplasm into 38 sub-populations. a) FineStructure pairwise coincidence, which represents the proportion of MCMC runs in which each pair of genotypes was clustered in the same sub-population; b) the FineStructure co-ancestry chunk counts for each pair of genotypes. Colors in the vertical legend represent genotype year of release (red: pre-1920, green: 1921 to 1970, and blue: post-1970); while horizontal legend colors represent the state of release (red: NSW, green: QLD, blue: SA, yellow: VIC, and cyan: WA).

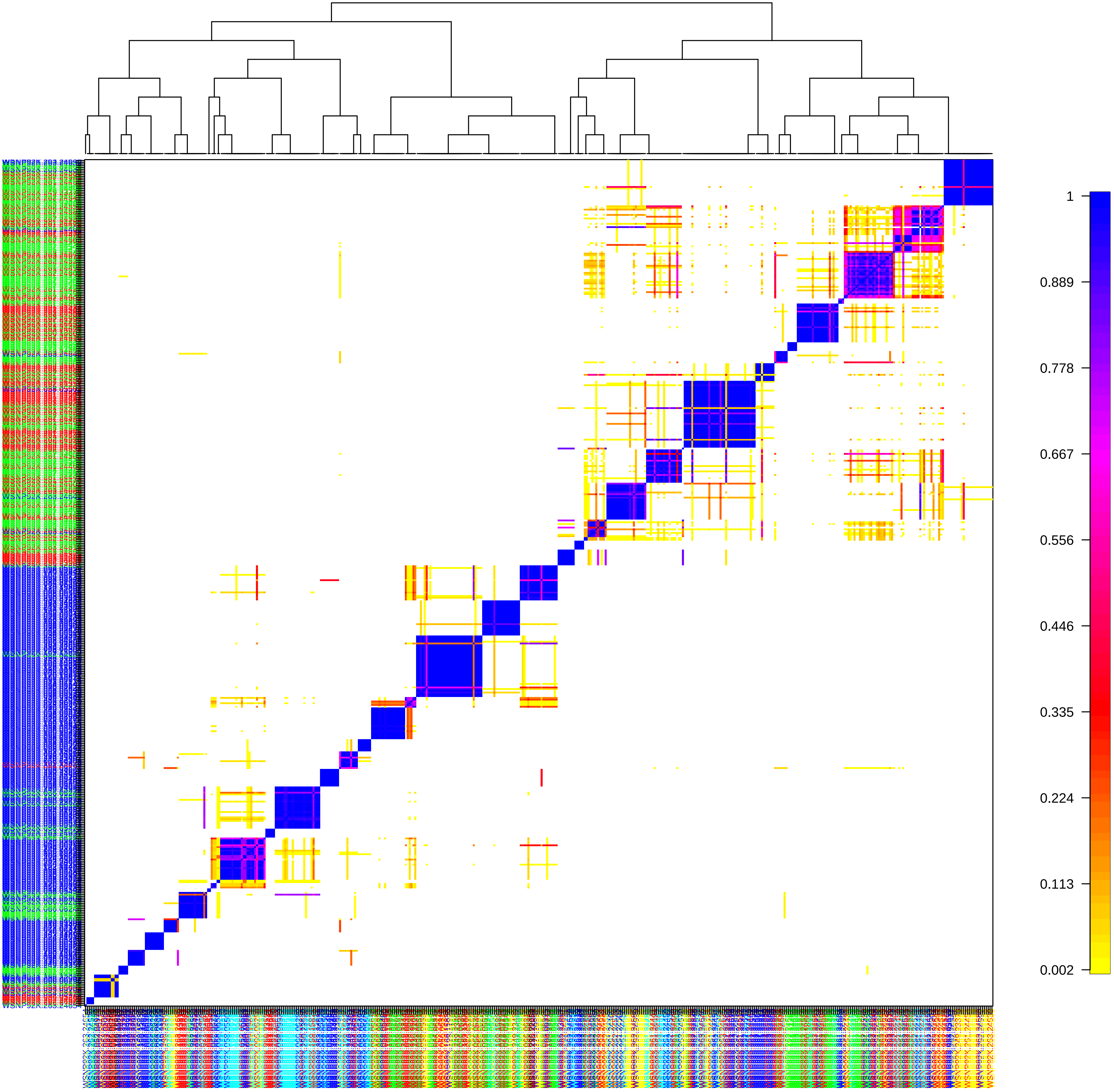
1
0.889
0.778
0.667
0.556
0.446
0.335
0.224
0.113
0.002

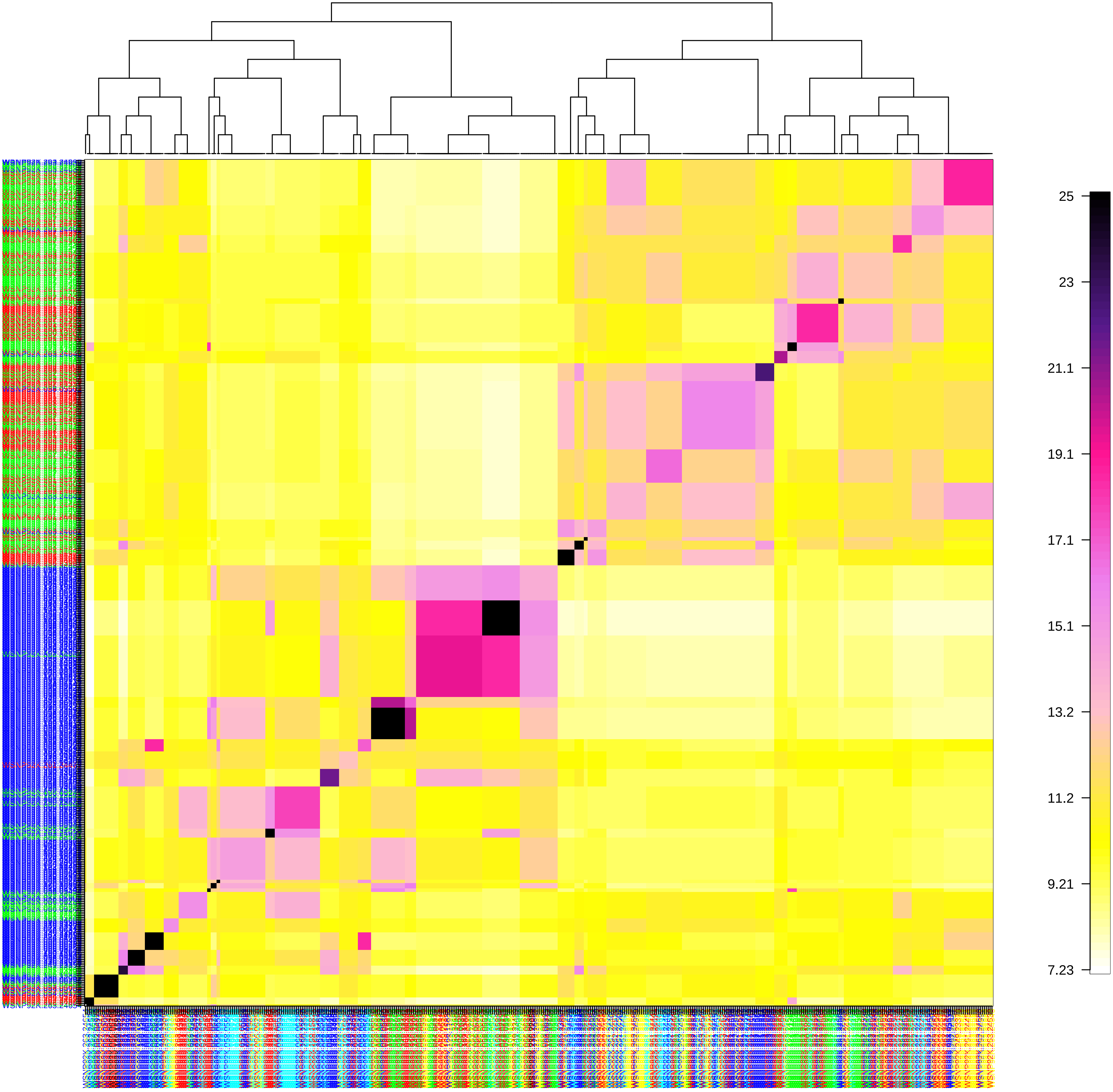
25
23
21.1
19.1
17.1
15.1
13.2
11.2
9.21
7.23
