# Supplementary material for: Genetic Diversity, Population Structure and Ancestral Origin of Australian Wheat
Source: Front Plant Sci. 2017 Dec 12;8:2115. doi: 10.3389/fpls.2017.02115 (PMC5733070; doi:10.3389/fpls.2017.02115)
Supplement: Supplementary file 4 [file Image4.PDF]

Figure S4. a) Bayesian Information Criterion (BIC) to define the optimal number of clusters revealed by k-means clustering ranging from 2 to 50; b) scatter plot of all Australian cultivars based on the DAPC output considering the *ADMIXTURE* grouping at K = 12.
Colors match the colors used for *ADMIXTURE* in figure 4, K=12.

a)

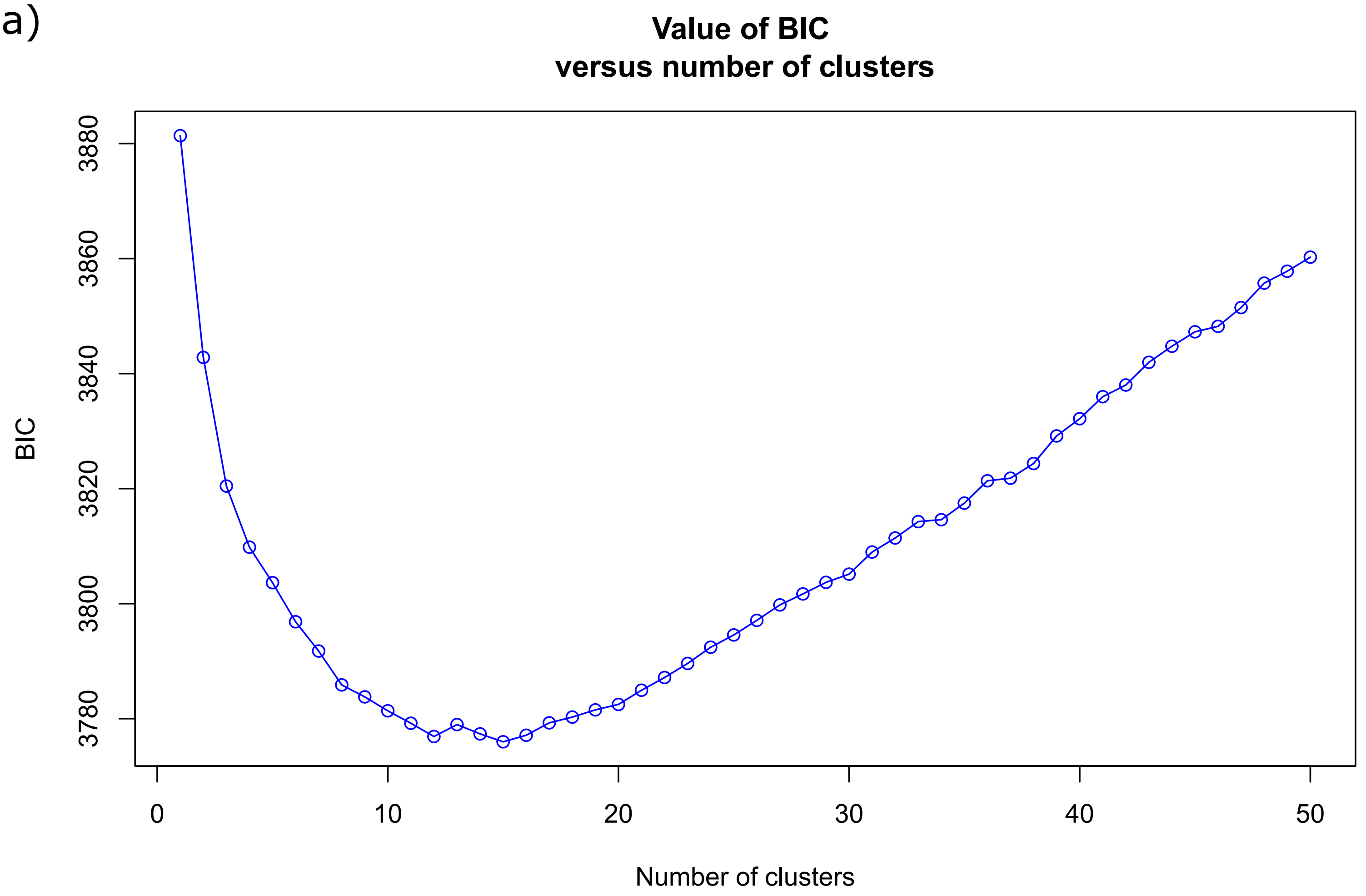

Value of BIC
versus number of clusters
BIC
3780
3800
3820
3840
3860
3880
0
10
20
30
40
50
Number of clusters

b)

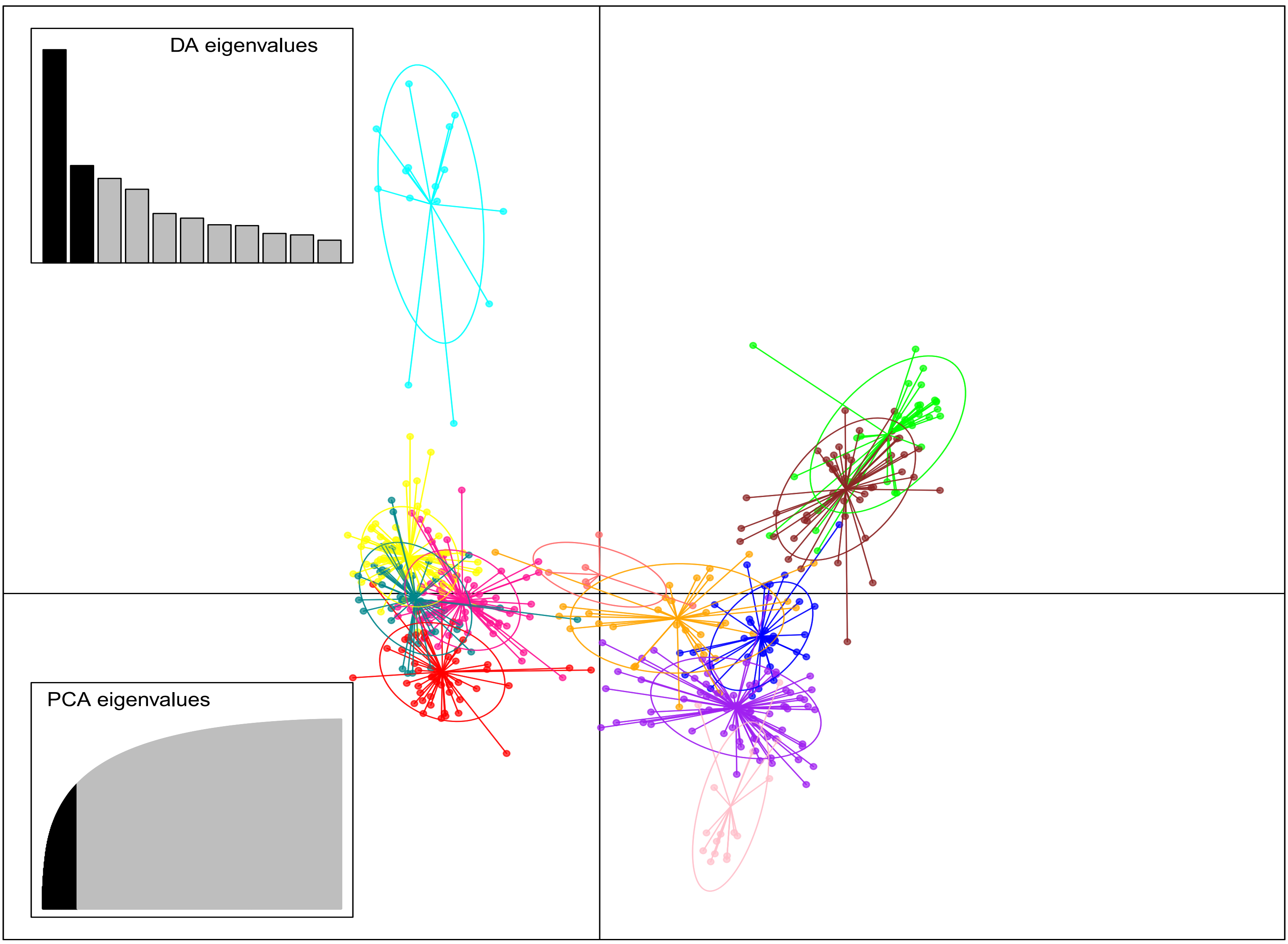

DA eigenvalues
PCA eigenvalues
