# Supplementary material for: Genetic Diversity, Population Structure and Ancestral Origin of Australian Wheat
Source: Front Plant Sci. 2017 Dec 12;8:2115. doi: 10.3389/fpls.2017.02115 (PMC5733070; doi:10.3389/fpls.2017.02115)

Figure S5. The ancestry proportions revealed by the *ADMIXTURE* analysis (K=12) for cultivars released in different Australian States. Colors match the colors used for *ADMIXTURE* in figure 4; K = 12.

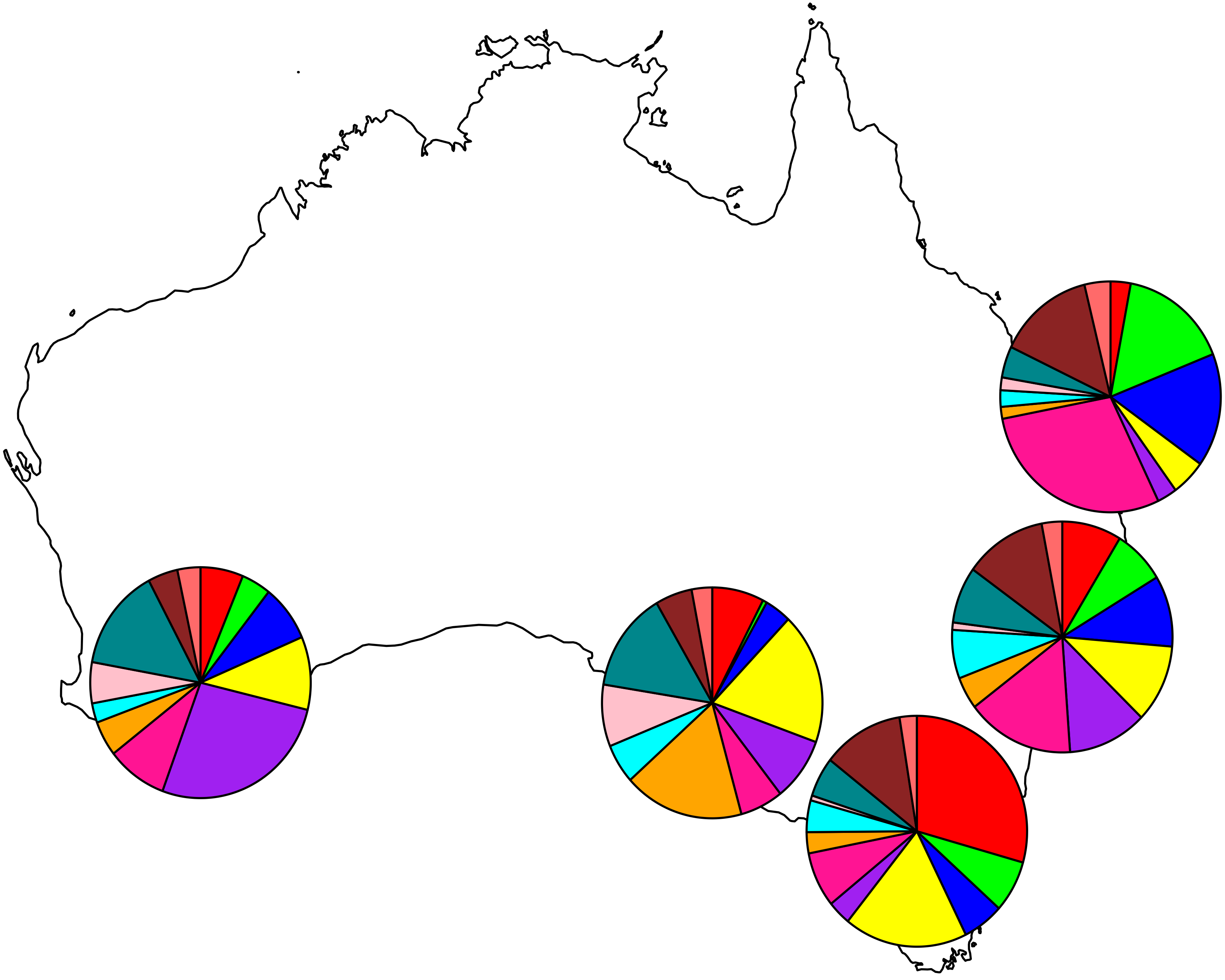

Supplement: Supplementary file 5 [file Image5.PDF]
