# Supplementary material for: Genetic Diversity, Population Structure and Ancestral Origin of Australian Wheat
Source: Front Plant Sci. 2017 Dec 12;8:2115. doi: 10.3389/fpls.2017.02115 (PMC5733070; doi:10.3389/fpls.2017.02115)
Supplement: Supplementary file 6 [file Image6.PDF]

Figure S6. *In silico* painting of the 482 cultivars representative of Australian germplasm divided by State and year of release. Each horizontal line represents one cultivar where the 21 chromosomes are ordered sequentially starting from chromosome 1A to 7D. NSW: New South Wales; QLD: Queensland; SA: South Australia; VIC: Victoria; WA: Western Australia. Colors describe worldwide populations following Fig. 1c.

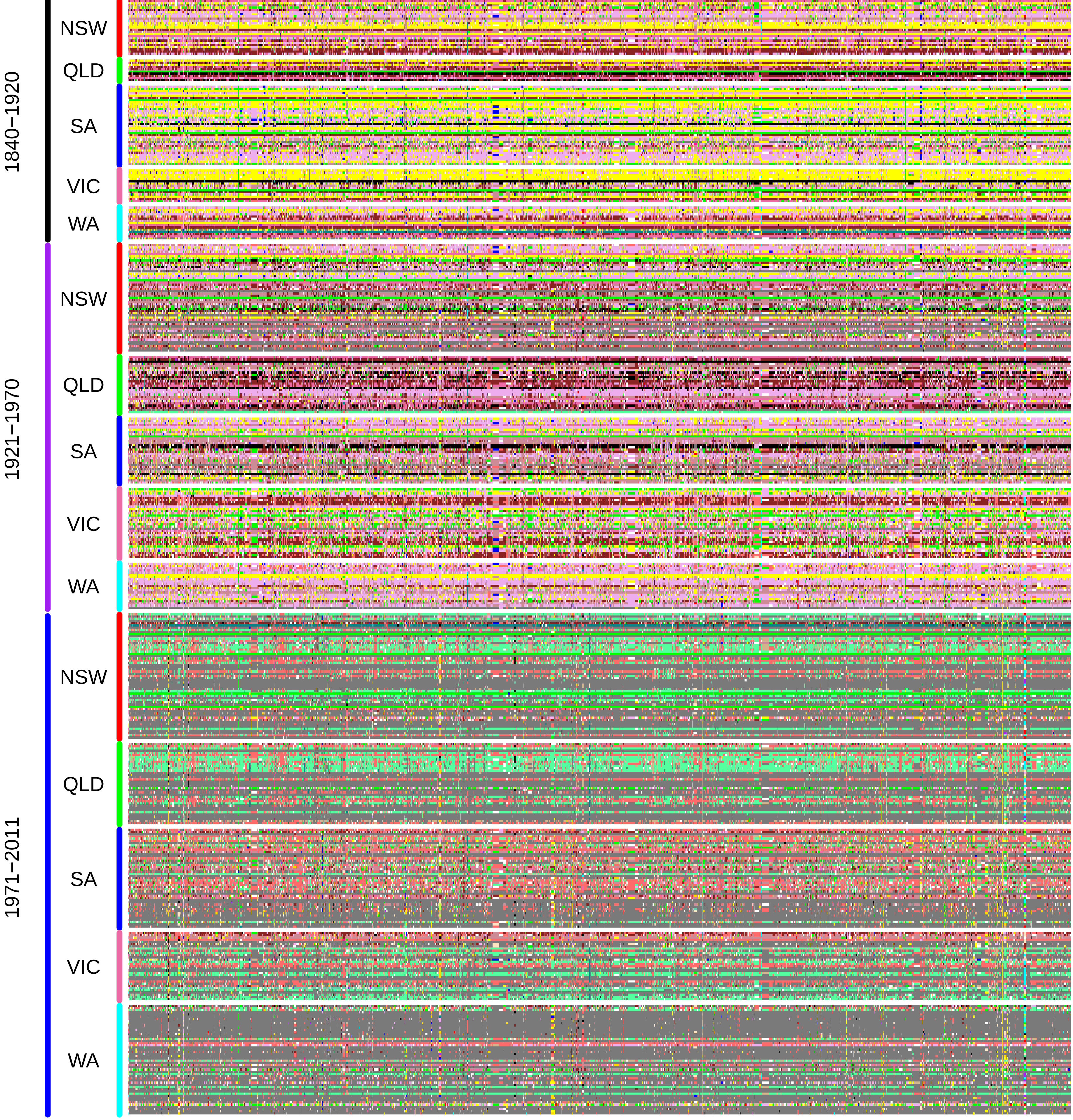
1840–1920
NSW
QLD
SA
VIC
WA
1921–1970
NSW
QLD
SA
VIC
WA
1971–2011
NSW
QLD
SA
VIC
WA
