# Supplementary material for: Genetic Diversity, Population Structure and Ancestral Origin of Australian Wheat
Source: Front Plant Sci. 2017 Dec 12;8:2115. doi: 10.3389/fpls.2017.02115 (PMC5733070; doi:10.3389/fpls.2017.02115)
Supplement: Supplementary file 7 [file Image7.PDF]

Figure S7. Overall contribution of each donor population to the ancestral makeup for cultivars released in each State in different time period. Colors describe worldwide populations following Fig. 1c.

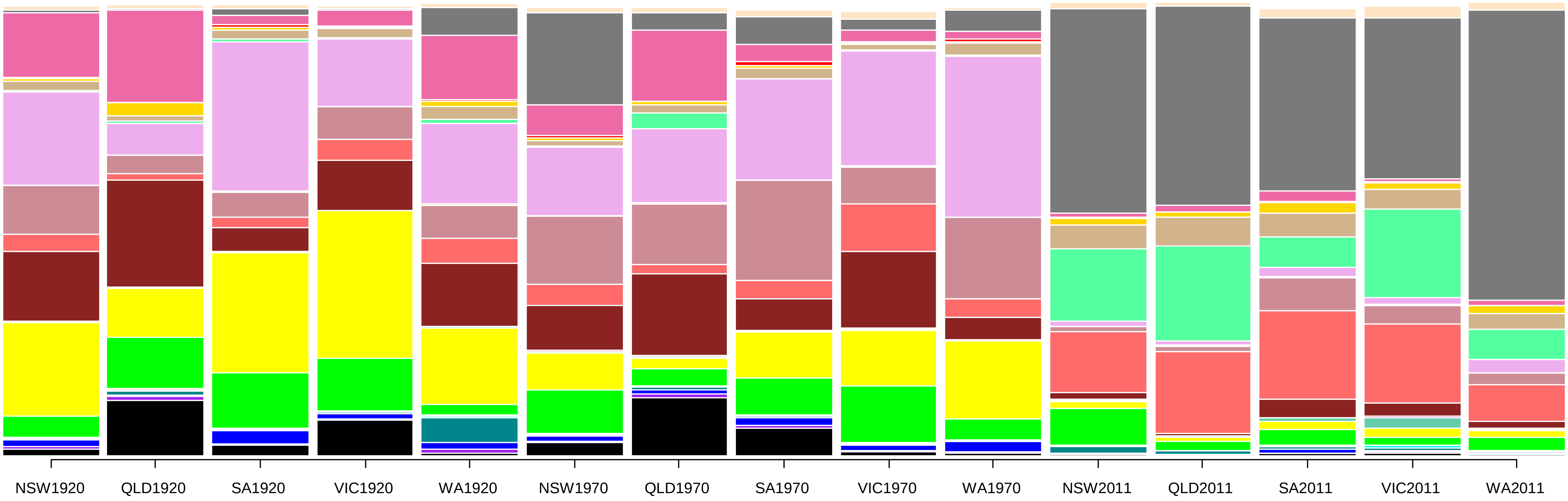

NSW1920
QLD1920
SA1920
VIC1920
WA1920
NSW1970
QLD1970
SA1970
VIC1970
WA1970
NSW2011
QLD2011
SA2011
VIC2011
WA2011
