# Supplementary material for: Genetic Diversity, Population Structure and Ancestral Origin of Australian Wheat
Source: Front Plant Sci. 2017 Dec 12;8:2115. doi: 10.3389/fpls.2017.02115 (PMC5733070; doi:10.3389/fpls.2017.02115)
Supplement: Supplementary file 8 [file Image8.PDF]

Figure S8. Expanding the ancestral makeup of chromosome 1B for cultivars released after 1970. The black box highlights the region of the translocation 1B/1R.

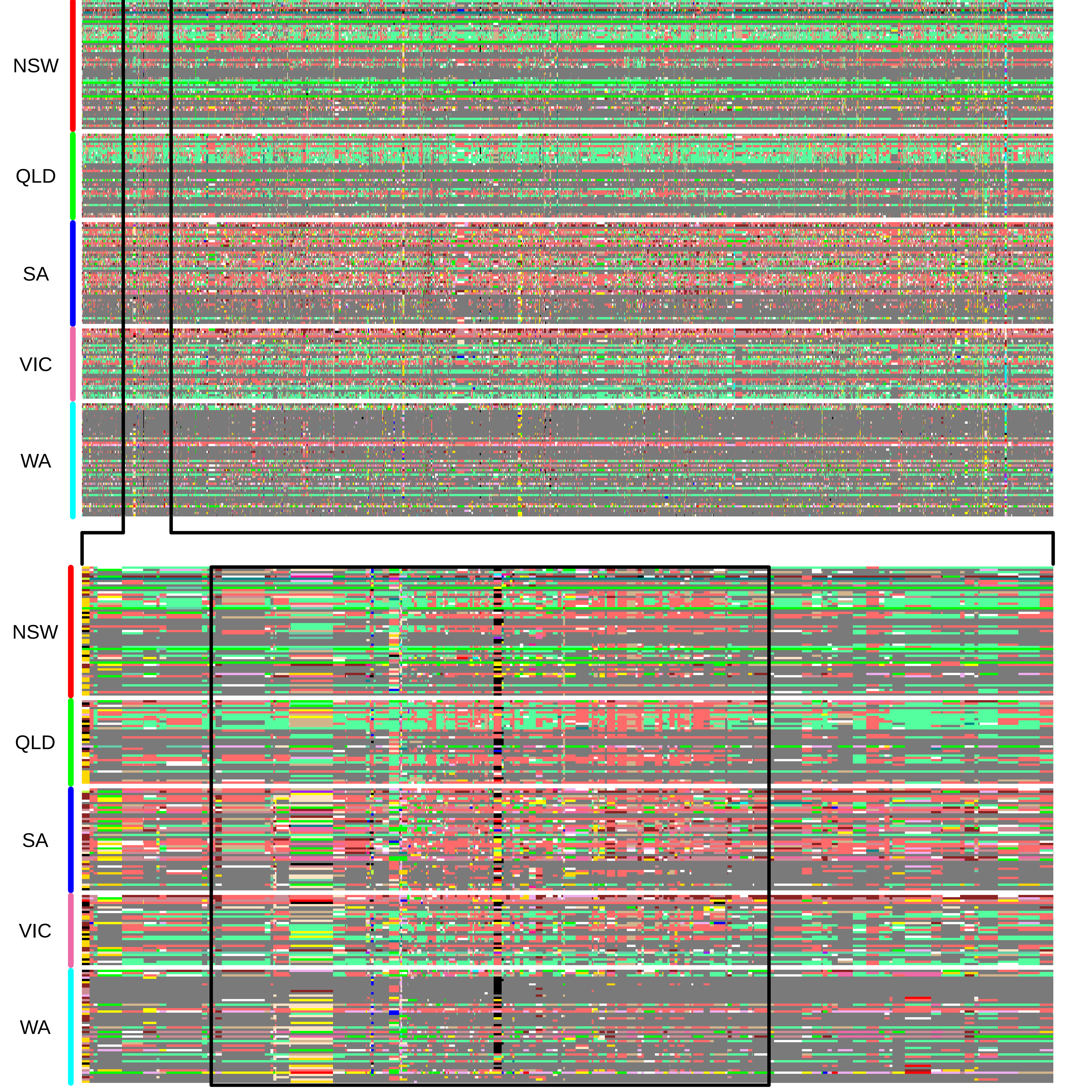
NSW
QLD
SA
VIC
WA
NSW
QLD
SA
VIC
WA
