# Supplementary material for: Genetic Diversity, Population Structure and Ancestral Origin of Australian Wheat
Source: Front Plant Sci. 2017 Dec 12;8:2115. doi: 10.3389/fpls.2017.02115 (PMC5733070; doi:10.3389/fpls.2017.02115)
Supplement: Supplementary file 9 [file Image9.PDF]

Figure S9. The ancestral makeup of selected Australian cultivars that have in their pedigrees a) Purple Straw; b) Red Fife; c) Kenyan cultivars; d) Gabo; and e) WW15.

Purple Straw

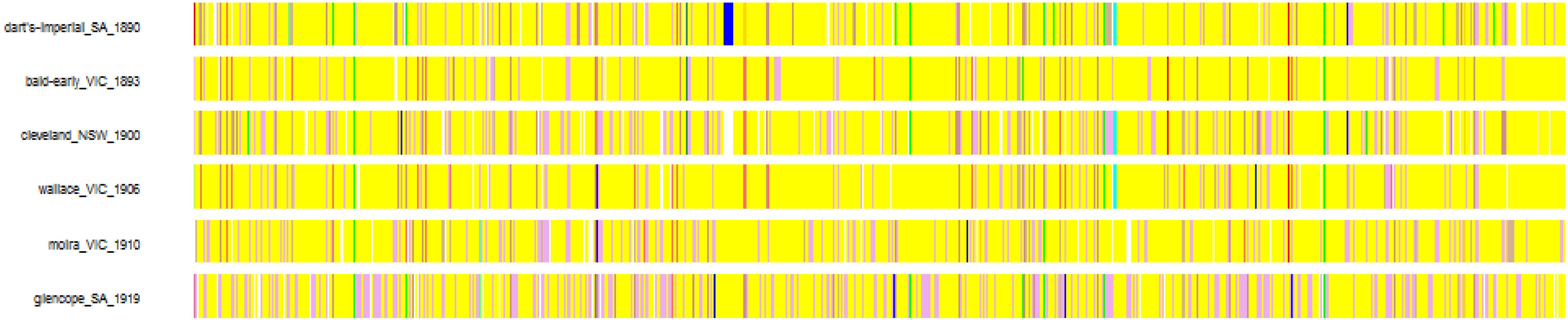

Red Fife

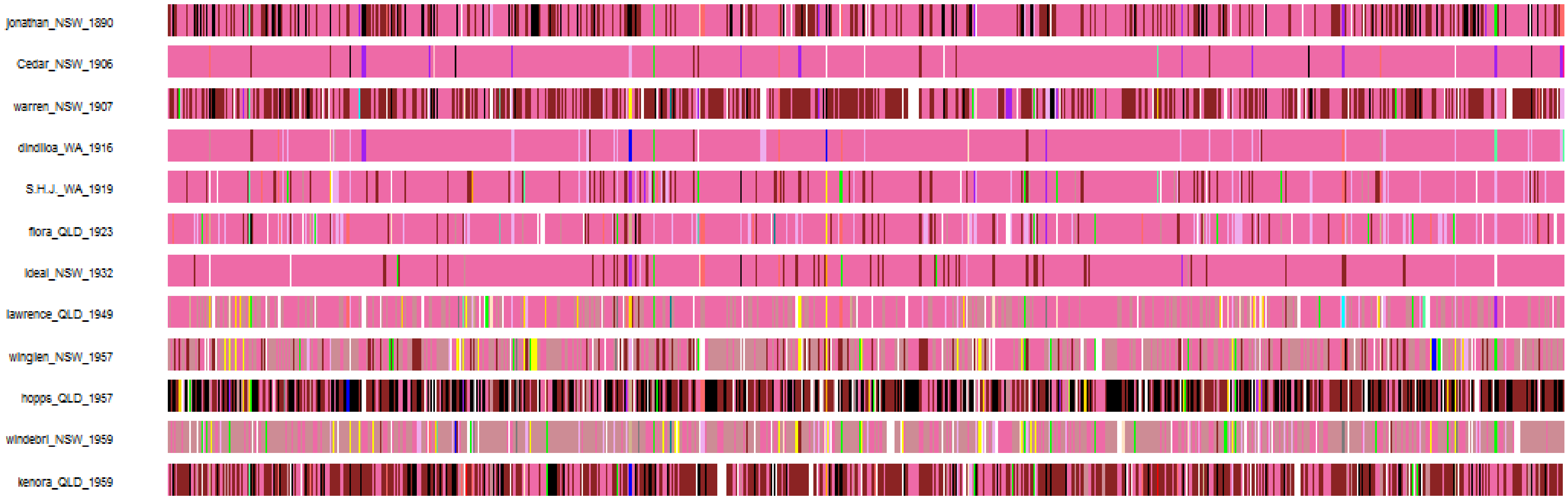
jonathan_NSW_1890
Cedar_NSW_1906
warren_NSW_1907
dindiloa_WA_1916
S.H.J._WA_1919
flora_QLD_1923
Ideal_NSW_1932
lawrence_QLD_1949
winglen_NSW_1957
hopps_QLD_1957
windebri_NSW_1959
kenora_QLD_1959

Kenyan accessions

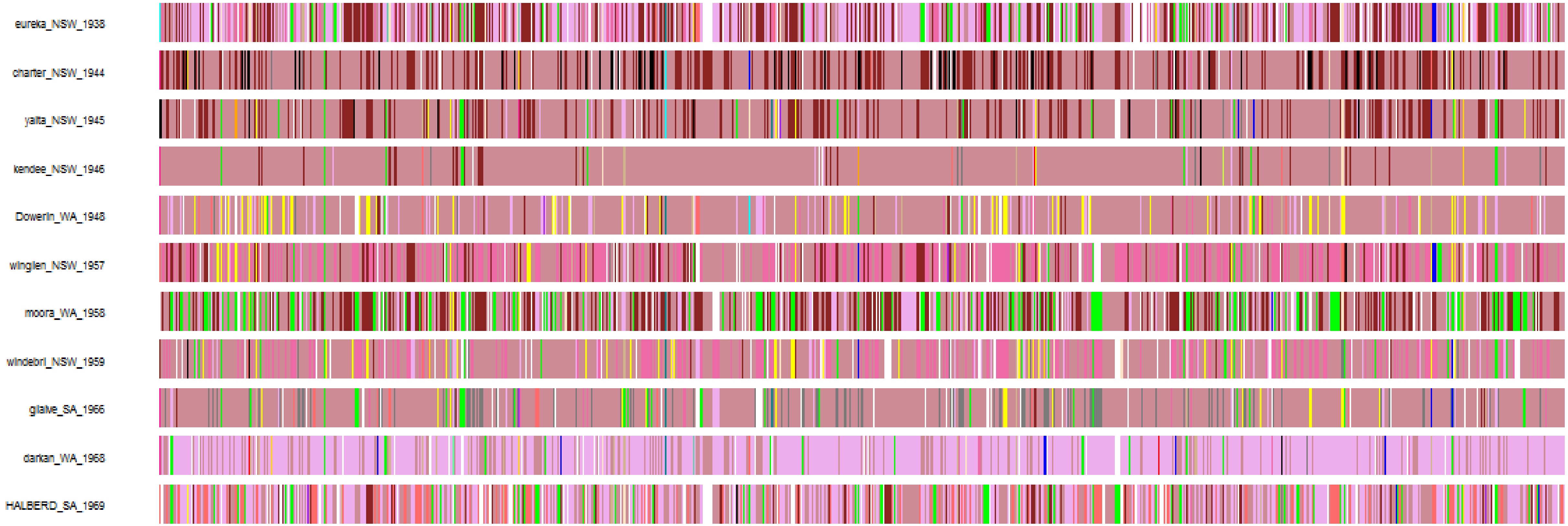

Gabo

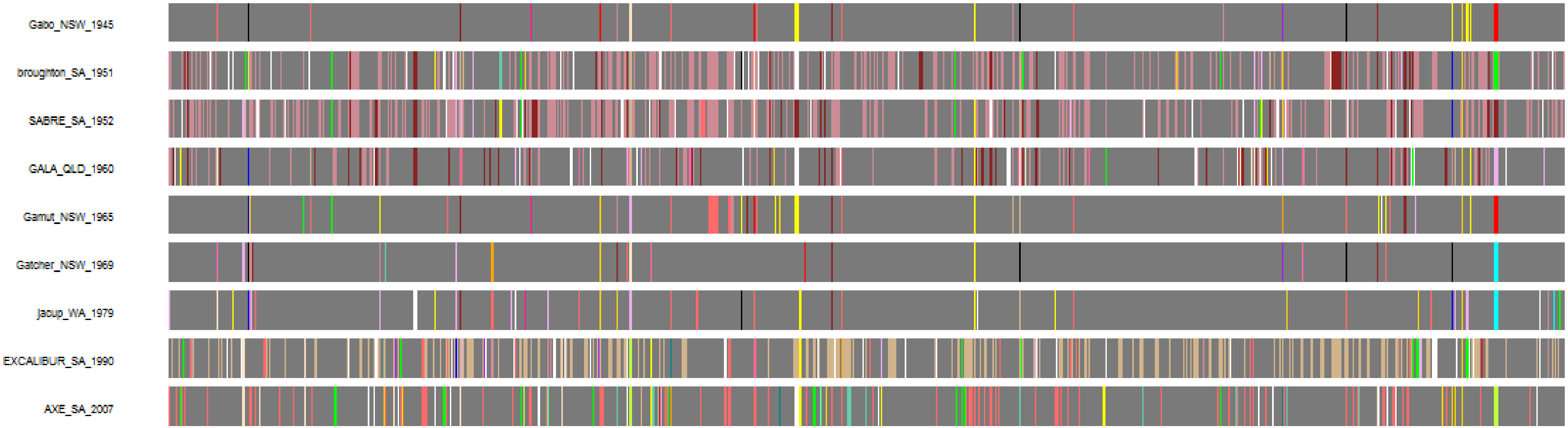
Gabo_NSW_1945
broughton_SA_1951
SABRE_SA_1952
GALA_QLD_1960
Gamut_NSW_1965
Gatcher_NSW_1969
jacup_WA_1979
EXCALIBUR_SA_1990
AXE_SA_2007

WW-15

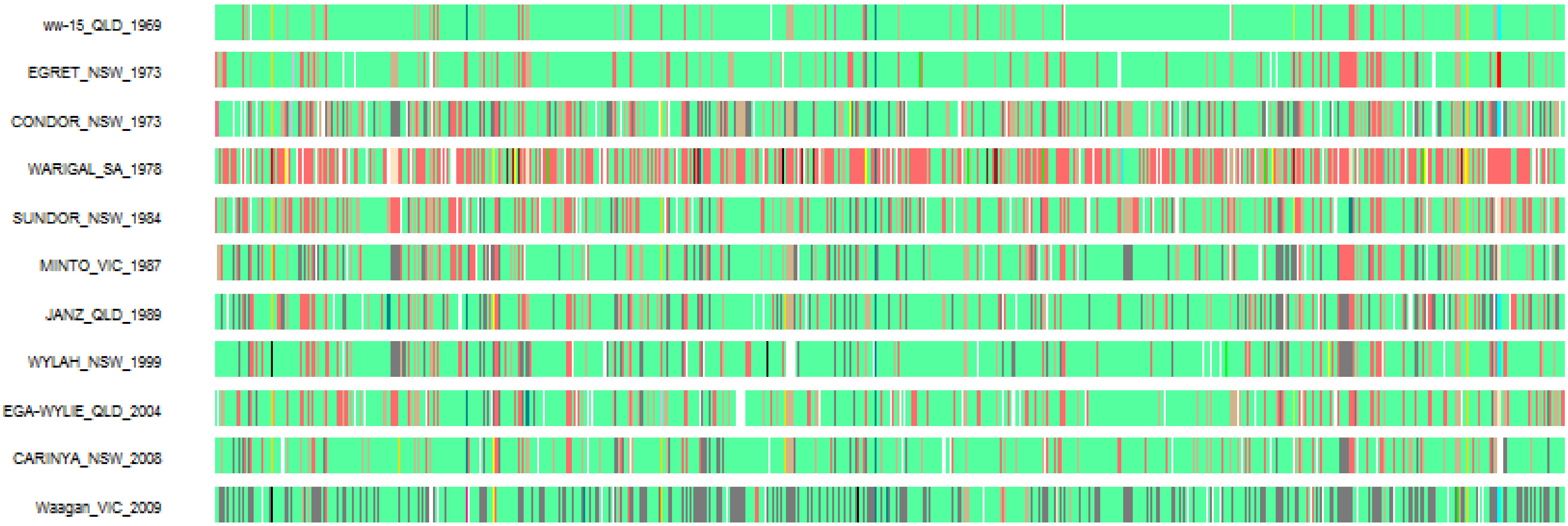
ww-15_QLD_1969
EGRET_NSW_1973
CONDOR_NSW_1973
WARIGAL_SA_1978
SUNDOR_NSW_1984
MINTO_VIC_1987
JANZ_QLD_1989
WYLAH_NSW_1999
EGA-WYLIE_QLD_2004
CARINYA_NSW_2008
Waagan_VIC_2009
