# Supplementary material for: Genetic Diversity, Population Structure and Ancestral Origin of Australian Wheat
Source: Front Plant Sci. 2017 Dec 12;8:2115. doi: 10.3389/fpls.2017.02115 (PMC5733070; doi:10.3389/fpls.2017.02115)
Supplement: Supplementary file 10 [file Image10.PDF]

Figure S10. *In silico* painting of the 482 cultivars representative of Australian germplasm divided by State and year of release after applying cleaner painting (removing the three African populations 11, 12 and 16). For details, see figure S6.

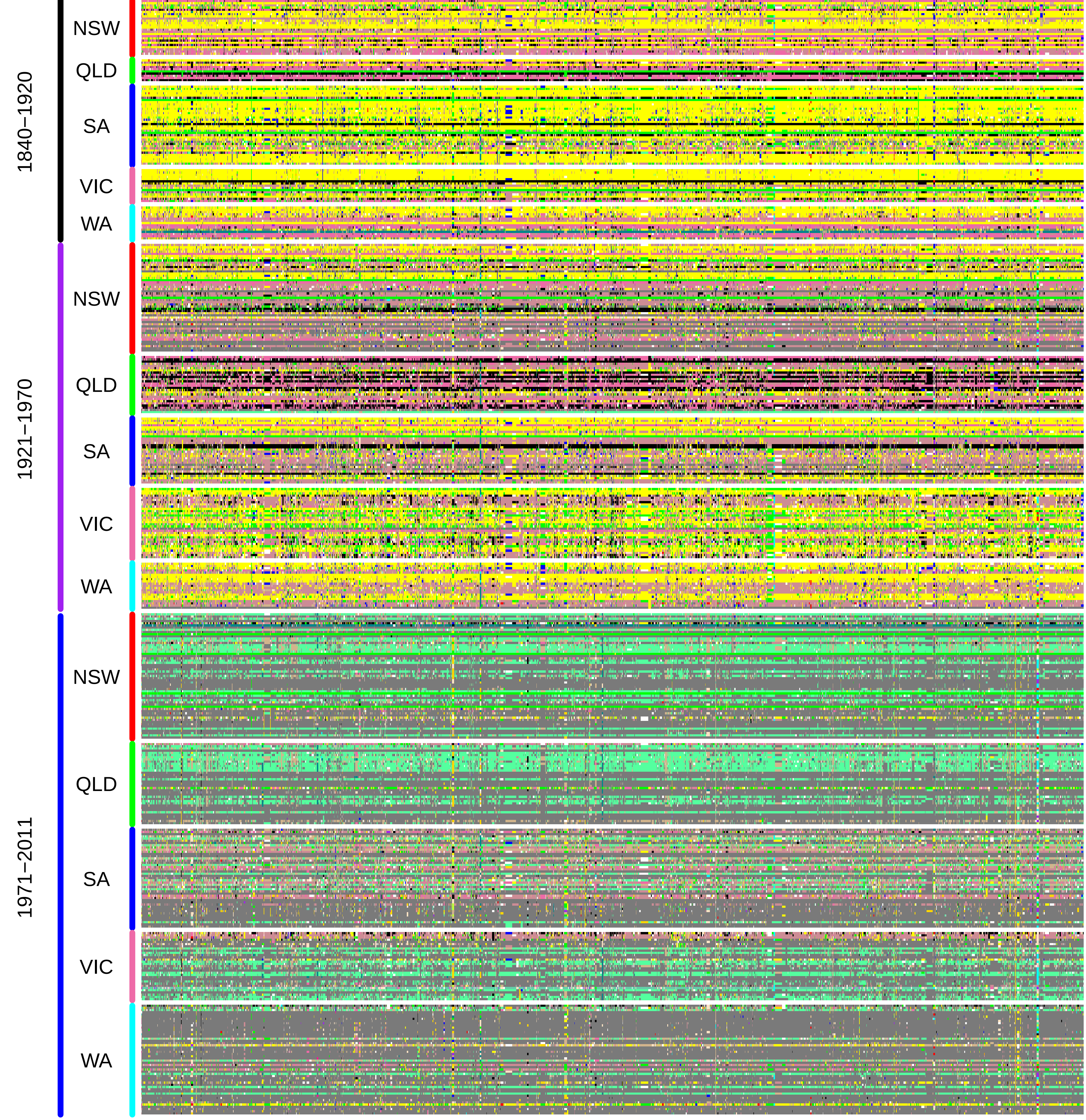
1840–1920
NSW
QLD
SA
VIC
WA
1921–1970
NSW
QLD
SA
VIC
WA
1971–2011
NSW
QLD
SA
VIC
WA
