# Supplementary material for: Genetic Diversity, Population Structure and Ancestral Origin of Australian Wheat
Source: Front Plant Sci. 2017 Dec 12;8:2115. doi: 10.3389/fpls.2017.02115 (PMC5733070; doi:10.3389/fpls.2017.02115)
Supplement: Supplementary file 11 [file Image11.PDF]

Figure S11. Proportion of ancestral contribution for each donor population at each SNP locus across the genome after applying cleaner painting. For details, see figure 5.

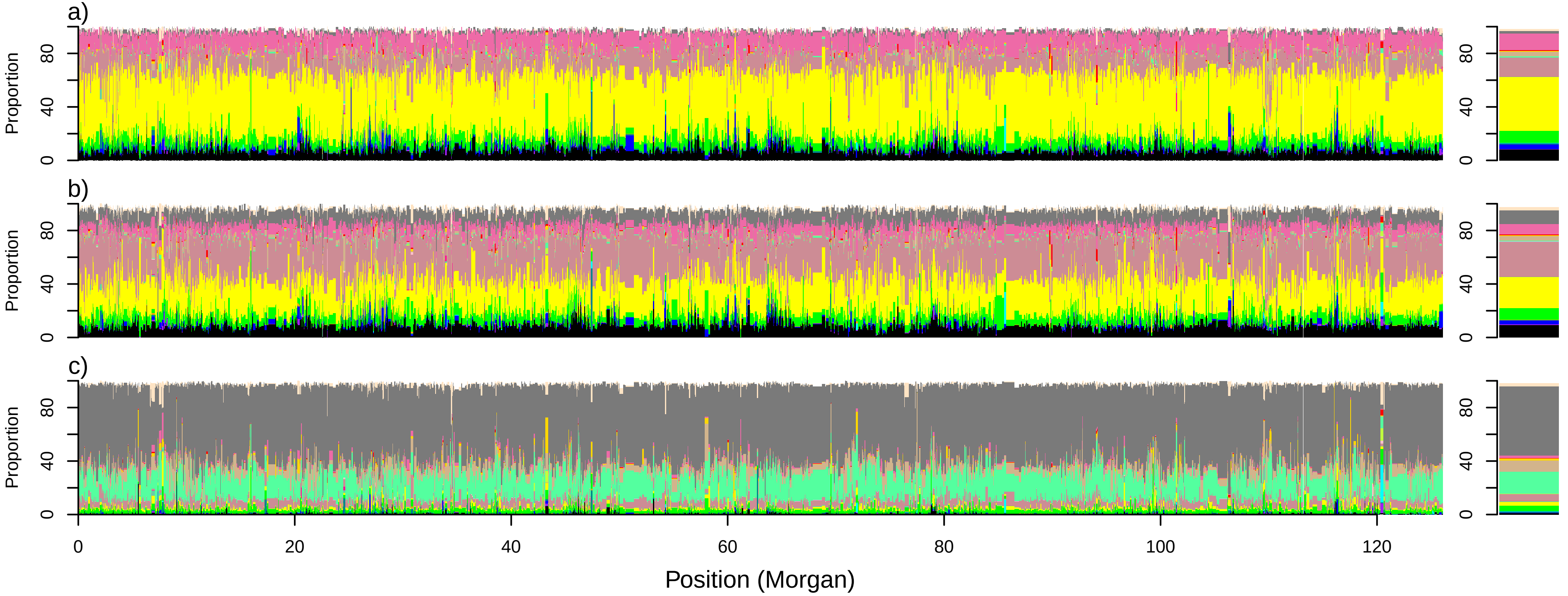

a)
b)
c)
Proportion
0
40
80
0
20
40
60
80
100
120
Position (Morgan)
